# Unveiling the role of novel biogenic functionalized CuFe hybrid nanocomposites in boosting anticancer, antimicrobial and biosorption activities

**DOI:** 10.1038/s41598-021-87363-z

**Published:** 2021-04-08

**Authors:** Marwa Eltarahony, Marwa Abu-Serie, Hesham Hamad, Sahar Zaki, Desouky Abd-El-Haleem

**Affiliations:** 1 Environmental Biotechnology Department, Genetic Engineering and Biotechnology Research Institute (GEBRI), City of Scientific Research and Technological Applications (SRTA-City), New Borg El-Arab City, Alexandria, 21934 Egypt; 2 Medical Biotechnology Department, Genetic Engineering and Biotechnology Research Institute, (GEBRI), City of Scientific Research and Technological Applications (SRTA-City), New Borg El-Arab City, Alexandria, 21934 Egypt; 3 Fabrication Technology Research Department, Advanced Technology and New Materials Research Institute (ATNMRI), City of Scientific Research and Technological Applications (SRTA-City), Alexandria, 21934 Egypt

**Keywords:** Applied microbiology, Environmental microbiology, Microbiology, Environmental sciences, Biotechnology, Environmental biotechnology

## Abstract

The quest for eco-friendly and biocompatible nanoparticles (NPs) is an urgent issue in the agenda of the scientific community and applied technology, which compressing synthesis routes. For the first time, a simple route for the biosynthesis of functionalized CuFe-hybrid nanocomposites (FCFNCs) was achieved using *Streptomyces cyaneofuscatus* through a simultaneous bioreduction strategy of Cu and Fe salts. The suitability of FCFNCs was evaluated medically and environmentally as an anticancer agent, antimicrobial agent and dye bio-sorbent. The physicochemical characteristics of FCFNCs using XRD, EDX, elemental mapping, FTIR, UV–Vis., TEM and ζ-potential confirmed the formation of spheres agglomerated into chains (37 ± 2.2 nm), self-functionalized nanocomposite by proteinaceous moieties with considerable stability (− 26.2 mV). As an anticancer agent, FCFNCs displayed the highest apoptotic impact (> 77.7%) on Caco-2, HepG-2, MCF-7 and PC-3 cancer cells at IC_50_ ≤ 17.21 μg/mL with the maximum up regulation of p53 and caspase 3 expression and the lowest Ki-67 level, relative to both functionalized CuNPs (FCNPs) and FeNPs (FFNPs). Meanwhile, it maintained the viability of normal human cells by EC_100_ up to 1999.7 μg/mL. Regarding the antimicrobial activity, FCFNCs offered > 70% growth reduction among wide spectrum prokaryotic and eukaryotic pathogens. Additionally, the synergistic feature of FCFNCs disintegrated the pre-established biofilm and algal growth in a dose-dependent manner. However, as a bio-sorbent, FCFNCs decolorized > 68% of malachite green and congo red dyes (200 mg/L), reflecting considerable remediation efficiency, confirmed by FTIR of FCFNCs- adsorbed dyes and microtoxicity/cytotoxicity of solutions after remediation. This study offers new insights into promising CuFe-hybrid nanocomposites for recruitment in several applications.

## Introduction

Miniaturization is an adopted phenomenon from nature since the evolution process until now, which considers being the fundamental principle of nanoscience and subsequent nanotechnological applications. Such naturally motivated technology addresses the art of designing materials in nanostructure configuration (less than 100 nm, at least in one dimension) that exhibit unique features surpassing their bulk-counterparts^1,2^. Over the recent decade, the paradigm leapfrogging in nanotechnology has led to an improvement in the methods of synthesizing nanomaterials that included the employment of green chemistry approaches^3,4^.

Such methods aimed to eradicate the use and production of hazardous substances that are detrimental to human health and the environment via an inexpensive, sustainable, eco-friendly, and innocuous means^5^. A plethora of bioresources available in nature including bacteria, yeast, fungi, algae, plants, and their derivatives mediate fabrication of nanostructures in biology-based green routs. They provide biomolecules that dually reduce the bulk molecules into their nanostructures and simultaneously cover their surface as a capping and dispersing layer which ultimately participate in their stability and biocompatibility^6^. Actinomycetes occupy a prominent position among prokaryotes at economic and biotechnological levels. This bacterial group characterized by the production of approximately 45% of bioactive secondary metabolites. Recently, it was categorized among natural nano factories by the dint of its capability to bind, accumulate, and reduce metals to their nanoforms through numerous, diverse, and valuable catalyzing and stabilizing biomolecules^7^.

Nowadays, nanocomposites offer greater interest from scientific as well as technological points of view for their distinctive structural and applicable features. Nanocomposite can be defined as a combination of materials of geometrical different architecture that exhibiting collective unique properties. Metal nanocomposites are assorted to different types, such as metal–metal, metal oxide–metal oxide, metal–metal oxide, metal–polymer and metal oxide–polymer^8^. The development of such new classes of nanomaterial was boosted to overcome the limitation of sole or mono-nanoparticles (NPs) and enhancing their properties for improved functionalities^8^.

Generally, the most extensively studied metals for such binary systems were Ag, Au, Pd and Pt^9,10^. However, the genotoxicity and cytotoxicity effects of these metals on humans and also their high cost limit their application, biologically in particular, as noted by Das et al.^11^ and Bakina et al.^12^. Therefore, it became necessary to substitute those metals with alternatives selected on the merits of safety, easy availability, catalytic properties, cost-effectiveness, reactivity, and overall performance as pointed out by Pinto et al.^13^. Copper (Cu) and iron (Fe) are naturally occurring micronutrients in the plant, animal and human tissues; they play vital roles in biochemical functions as synthesis of the DNA, cell wall metabolism, oxidative stress and respiration. Remarkably, Fe-based nanoparticles (FeNPs) gained approval from FDA (US Food and Drug Administration) for use in several sectors, including magnetic resonance imaging for liver pathologies detection, iron deficiency anemia therapy in adults with chronic kidney disease and in water purification process^11,14^. In the same context, Cu-based nanoparticles (CuNPs) were permitted by EPA (US Environmental Protection Agency) as an antimicrobial agent^11^. Hence, by the dint of their efficiency, low toxicity, safety and biocompatibility, both fell in our circle of interest. Notably, the heterozygosity of both metals and their oxides in hybrid configuration was comprehensively documented in versatile applications, such as microbial disinfection^4,11,12,15^, phenolic compounds degradation^16^, heavy metals removal^17^, photocatalysis^18^**,** higher alcohol production^19^ and wound healing^20^.

While there is a remarkable plurality of physicochemical routs such as chemical reduction^19^, ball milling^11^, spray pyrolysis^21^, etc. for the preparation of such bifunctional systems, the biogenic approach to synthesize such hybrid NPs is restricted to Phytoextracts^3^. As compared with the physicochemical routes, many more difficulties encounter microbially-assisted preparation of hybrid systems of metal/metal oxides nanocomposites, which is hardly examined as stated by Khatami et al.^3^ and Omajali et al.^10^. Where, uncontrolled behavior of microbe in metal selectivity and its response against the dual coexistence of metals are obscured. Besides, the metabolic pathway conducted either successive or concurrent metals reduction, intracellularly or extracellularly; the probability of employing mutual mechanisms during the reduction process such as precipitation, bioaccumulation, biomineralization, and biosorption are also included. These reasons and more pose questions, namely, which dually architecture among homogeneous alloy, core–shell, Janus, multi-shell or mixed monometallic would be produced? What would be the chemical composition and percentage of each metal in the resulting nanocomposite? Which size or morphology could be resulted? Could the uniformity and dispersity be controlled? Could the variation in the molar ratio of the individual metal provide a new dimension in tailoring the features of nanocomposite? Could the microbial cultural conditions influence surface energies, electronegativity, atomic size and oxidation potential of binary metals? Finally, could an unexpected ordering pattern of binary NPs enhance the properties of nanocomposite to exceed their mono-structure NPs?

In light of the previous, this investigation focused on the bacterial synthesis of functionalized CuFe-based nanocomposites (FCFNCs) using actinomycetes isolate in the simultaneous presence of Cu and Fe salts. The as-prepared FCFNCs were characterized structurally and morphologically. The proposed mechanism for production was also clarified. Consequently, the biological activity, in the form of cytotoxicity, anticancer capability and antimicrobial potency was determined; in addition, the behavior of FCFNCs in dye removal was evaluated as well. To the best of the authors' knowledge, no study has so far been recorded concerning the employment of *S. cyaneofuscatus* in the preparation of FCFNCs in a simple and cost-effective method for medical and environmental applications.

## Results and discussion

### Biosynthesis and physicochemical characterization of NPs

The biosynthesis of functionalized CuNPs (FCNPs) was initially validated by change of the color of *S. cyaneofuscatus* EM3 culture from pale green to faint brown. On the other hand, *S. cyaneofuscatus* EM3 culture containing functionalized FeNPs (FFNPs) and FCFNCs exhibited dark brown to black color. Comparatively, no obvious change of color occurred in the control samples, implying successful bioconversion of metal precursors into their corresponding NPs. The characteristic features and structural verification of biologically prepared NPs were elaborately evidenced with the following analysis:

#### Structural characteristics

**Figure 1:**
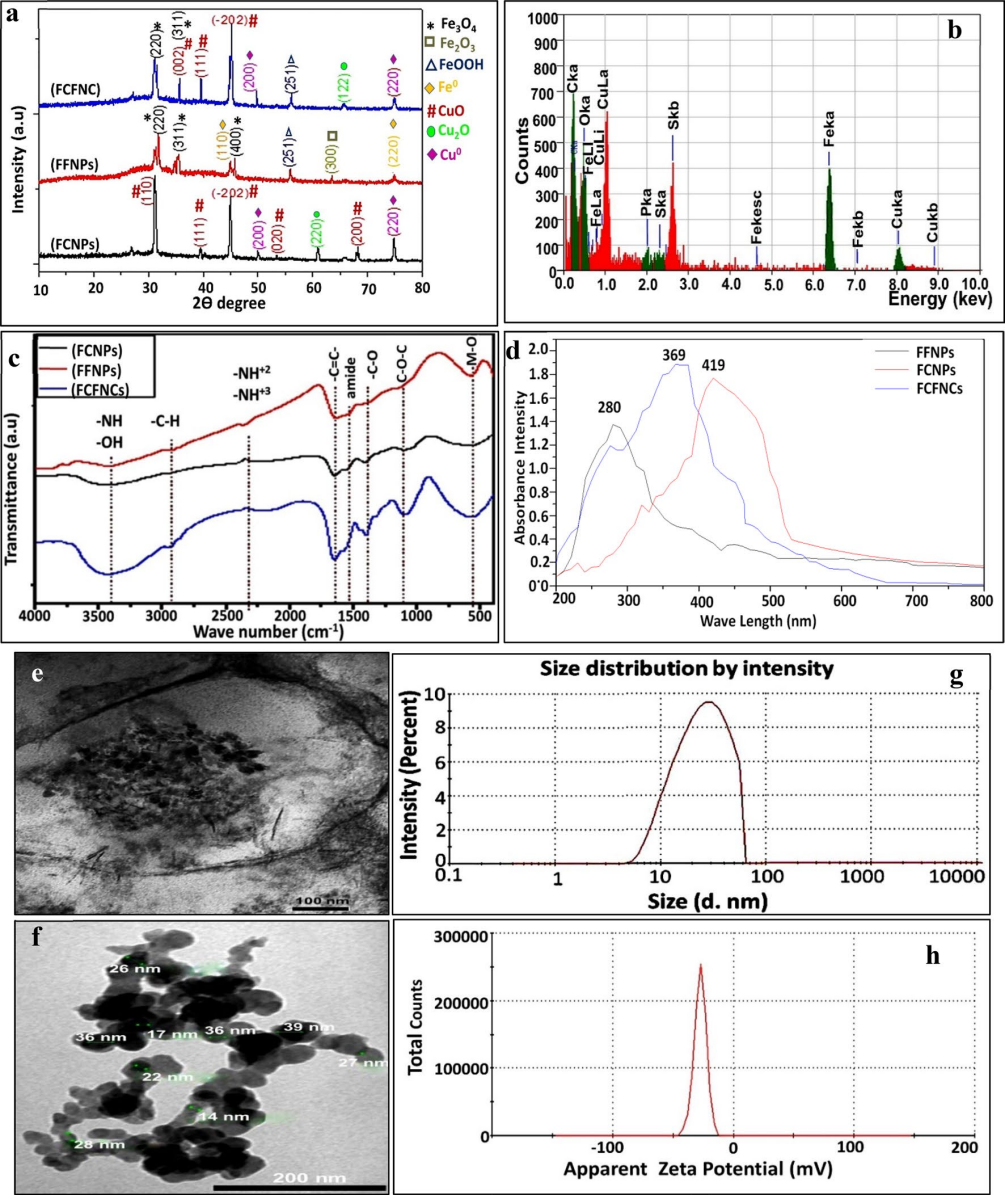
Characteristic features of the biosynthesized FCFNCs; XRD (**a**), EDX (**b**), FTIR (**c**), UV–Vis (**d**), TEM micrograph of cytoplasmic-localized FCFNCs synthesized by *S. cyaneofuscatus* EM3 during stationary phase (**e**), after extraction (**f**), Particle size distribution curve (**g**) and Zeta potential (**h**).

The crystalline structure and phase identification of FCNPs, FFNPs and FCFNCs were determined through X-ray diffraction (XRD) (Fig. 1a) and their Joint Committee on Powder Diffraction Standards (JCPDS) (Supplementary Fig. S1). As a general observation, the sharp and distinct peaks in XRD diffractograms revealed the crystalline nature of biosynthesized NPs with the lowest surface energy^22^. However, other biologically or even chemically synthesized NPs signified the production of imperfect crystals or amorphous phase^9,23^. The diffractogram of FCNPs showed characteristic peaks at 2θ = 30.9°, 39.42°, 44.82°, 53.53°, and 68.38° which correspond to (110), (111), (− 202), (020), and (200), Bragg’s reflection, respectively. These diffraction planes were matched with values of the monoclinic cubic phase of CuO (JCPDS file no. 00-005-0661)^24,25^. The peak at 2θ = 60.95° (220) indexed cubic structure of Cu_2_O-NPs (JCPDS file no. 00-005-0667) as highlighted by Zhao et al.^26^. In addition, the presence of diffraction peaks at 49.9° and 74.89° matched reflection from (200) and (220) planes of the pure face-centered cubic structure of Cu^0^ (JCPDS file no. 00-004-0836)^24^. Obviously, the main phase was CuO with low amounts of Cu_2_O phase in the examined sample of FCNPs. Notably, the oxidation states of Cu in the examined sample, including (0), (+ 1), and (+ 2), were very typical. The position of these peaks matched well the reports by Zhao et al.^26^ and Karimzadeh et al.^27^, suggesting that Cu/Cu_2_O/CuO composites were successfully synthesized in the same sample.

On the other hand, the crystalline structure of the as-prepared FFNPs exhibited Fe^0^/FeOOH/Fe_2_O_3_/Fe_3_O_4_ as shown in Fig. 1a. The formed hexagonal Fe_2_O_3_ and cubic Fe_3_O_4_ nanostructures within the designed nanocomposite were indexed by (JCPDS file no. 01-89-0596] and (JCPDS file no. 01-071-6336], respectively^28,29^. The peak at 35.61° might be a combination of Fe_2_O_3_ and Fe_3_O_4_. The peak at 45.02° and 74.8° matched the reflection from (110) and (220) planes confirming the formation of cubic Fe^0^ based on the (JCPDS file no. 01-071-4649)^30^. Diffraction peak at 55.9° was assigned to the reflection from (251) plane of β-FeOOH^31^. Apparently, the polymorphic phase with heterostructure nanoparticles was produced via *S. cyaneofuscatus* EM3. These results revealed the synthesis of various crystalline types of the copper and iron derivates within the nanocomposite.

Notably, the XRD pattern of FCFNCs confirmed the formation of a hybrid composite of Fe_3_O_4_/FeOOH/Cu_2_O/CuO/Cu with a predominance of Fe_3_O_4_ and CuO indicated by the existence of their distinctive peaks. Thus, such result proposed that *S. cyaneofuscatus* EM3 mediated the synthesis of FCNPs, FFNPs and FCFNCs in mixed phases of metallic and oxidized states. The detection of oxides in metallic phase NPs is a well-known phenomenon that could be ascribed to the surface oxidation of metallic NPs during synthesis, processing, drying and XRD-preparation steps. This finding agreed with other studies^9,32^. The absence or weakening of some distinctive peaks of Cu, FeOOH, and Cu_2_O in FCFNCs revealed the low content and poor nature of crystallinity^17,33^. More so, the presence of a small background hump at 2θ range 20°–30° could be assigned to bacterial organic biomolecules associated with the NPs, which was frequently observed in green protocols and also reflected the successful functionalization with metal/metal oxides. That was supported by FT-IR as described below^6,23^^,^^32^.

#### Compositional characteristics

The elemental distribution of iron and copper in the examined FCFNCs was proved through mapping and energy dispersive X-ray spectrometry (EDX). As shown in Supplementary Fig. S2, the elemental mapping of FCFNCs referred to the presence of Cu, Fe, and O entities in a similar and homogeneous distribution, which deducing binary hybrid structure material. However, Fig. 1b illustrated three major peaks of Cu, Fe and O in the FCFNCs sample with weight percentages of around 33.1%, 30.6% and 29.5%, respectively. Accordingly, the EDX profile determined the atomic proportion of Cu:Fe (1:0.92) in the FCFNCs which was in consonance with the inoculated molar ratio (1:1) of the precursors Cu(NO_3_)_2_ and Fe(NO_3_)_3_·9H_2_O. Concerning EDX profiling of FCNPs and FFNPs, intense signals of Cu, Fe and O were observed at surface energies corresponding to 6.4 keV, 8 keV, and 0.5 keV, respectively (Supplementary Fig. S3). Additionally, the signals related to C, P and S at 0.27, 2.0 and 2.3 keV, correspondingly were significantly detected in the EDX spectrum. That could be assigned to the X-ray emission from the proteinaceous and nuclear moieties associated with as-synthesized NPs. This combination could furnish NPs with stability by acting as a capping agent. Thus, such self-functionalization property provided by the biological method in one step with a reduction during the biosynthesis process is considered indigenously behaved and replaces the additional surface modification step, that is critically required for coating chemically and physically synthesized NPs^2^. The involvement of these elements with NPs was commonly reported particularly with green approaches^34^.

#### Functional characteristics

Fourier-transform infrared spectroscopy (FTIR) measurements provided presumptive insight into the surface chemistry of biosynthesized NPs by identifying the functional groups of microbial biomolecules conjugated with them, which caused their biosynthesis and stabilization. Figure 1c and Table 1 manifested the presence of common bands in all examined samples, which coincided with the results of^12,35^. Regarding the fingerprint region, the vibrational modes for FCNPs and FFNPs were vividly displayed at wavenumbers 547 cm^−1^ and 583 cm^−1^ which belong, respectively, to Cu–O and Fe–O vibration stretching. These results agreed with He et al.^33^ and Pandharipande and Makode^35^. In general, in view of internuclear vibrations, the metal oxide absorption peaks were observed at a lower field in range the 400–700 cm^−1^, which reflected the metallic nature of any examined sample^7,12,36^. However, small shift in the absorption peaks was observed in FCFNCs profile which may be due to the interaction between CuNPs and FeNPs and also the association of FCFNCs with carboxylic acids in the protein, which led to functionalization and dispersion of FCFNCs as implied by Ismail et al.^6^. Virtually, the association of several functional groups like C=O, C=C, C–O–C, PO_4_^3−^, amine and amide with NPs seemed to be advantageous^4,6,37^.

**Table 1:** FTIR peaks assignments and their corresponding functional groups associated with FCNPs, FFNPs and FCFNCs.

Wave number (cm^−1^)	Vibration type	Assignment
3700–4000	Stretching	O–H group
3440	Stretching	NH_2_
3434	Stretching	O–H group
2966	Stretching	C–H
2198–2391	Stretching	NH^2+^ and NH^3+^ in protein/peptide bonds
1648–1652	Stretching	–C=C bond
1567	Stretching	Amide II
1500–1700	Stretching	Amide I/II groups
1401–1414	Stretching	C–O
1452	Stretching	C=O of carboxylic acid
1250	Symmetric stretching	C–O–C
1200–1250	Symmetrical stretching	PO^2−^
1078	Stretching	To C–N of amines
1150–950	Stretching	PO_4_^3−^
1050–1100	Stretching/asymmetric stretching	C–O/C–O–C

#### Optical characteristics

The optical and electronic structure of FCNPs, FFNPs, and its combined hybrid system FCFNCs were scrutinized by UV–Vis spectroscopy. As depicted in Fig. 1d, a single surface plasmon resonance (SPR) band was observed at 280 nm and 419 nm for FCNPs and FFNPs, respectively. Similar results were found elsewhere^2,4^. Remarkably, the SPR band is considered a special and unique property for materials of metallic nature; nevertheless, it is sensitive to surface modifications and varies with solution chemistry and synthesis method^4^. On the other hand, a relative change in the absorbance maxima of the combined hybrid system FCFNCs appeared at 369 nm, which was an intermediate location between the SPR band of FCNPs and FFNPs. As asserted by Banik et al.^9^, UV–Vis spectroscopy is a valuable tool to signal the architecture attained by any binary or hybrid NP, as a randomly organized hybrid metal/metal oxides identified by a single SPR peak at an intermediate position between the wavelengths of the two metals. Likewise, Thakore et al.^38^ and Al-Asfar et al.^39^ recorded the difference in optical responses due to the variation of the structural features derived from hybrid mixing of Ag/Cu and Ag @ Fe, respectively.

#### Morphological characteristics

The morphology, interior structure and textural properties of as-prepared NPs were visualized by transmission electron microscopy (TEM). Figure 1e and Supplementary Fig. S4a and c illustrated the nanofactory *S. cyaneofuscatus* EM3 cell encompassing NPs; Fig. 1f and Supplementary Fig. S4b and d showed the extracted NPs. The TEM micrographs showed heterogeneity of the shapes and sizes of the FCNPs and FFNPs. The FCNPs synthesized intercellularly by *S. cyaneofuscatus* EM3 displayed needle- or rod-like morphology, more or less of similar size (12–36 nm) and homogeneous distribution without aggregation (Supplementary Fig. S4a). Whereas the extracted FCNPs appeared aggregated in nanoclusters and embedded in protein microbial matrix as illustrated by elongated dark NPs in the less dense light matrix (Supplementary Fig. S4b). Such microbial matrix provided stability to NPs and functionalized them as was previously indicated by EDX and FTIR. Regarding FFNPs, *S. cyaneofuscatus* EM3 formed numerous, tiny, quasi-spherical or roughly globular NPs with size oscillated between 2 and 7 nm and showed a slight tendency for agglomeration (Supplementary Fig. S4c). The extracted FFNPs presented well separated, well-defined, monodispersed spheres (Supplementary Fig. S4d). Such disparity among the morphologies of FCNPs and FFNPs and even FCFNCs was observed and reported earlier by Nadeem et al.^40^, who ascribed that to metal type, microbial chemistry, selective interaction mechanism and the overall physical conditions of the mixture.

However, FCFNCs exhibited undefined, irregular shapes and assembled in bulks or nanoclusters, appearing as electron opaque spots (Fig. 1e). Besides, a small percent of rods was also observed which could be attributed to Ostwald ripening mechanism^41^. Upon extraction, FCFNCs seemed mostly spheres of 37 ± 2.2 nm, not well-separated from each other and agglomerated occasionally into chains (Fig. 1f). Such chain like FCFNCs were previously found by Sepúlveda et al.^16^ and Shubair et al.^42^, who attributed this aspect to the higher surface energy of individual Fe-NPs (2.63 eV/atom) than Cu-NPs (1.37 eV/atom), the physicochemical characterization of the sample and the higher stability of NPs of such morphology. The aggregated behavior of FCFNCs could be attributed to the magnetic forces, electrostatic attractions among NPs, surface energy differences between Cu/Fe and the presence of H-bonding in the microbial bioactive molecules associated with the NPs^1,32^. Interestingly, the size and morphology of FCFNCs in our study coordinated with those prepared by Tabrizian^32^. Furthermore, the particle size distribution (PSD) curve of FCFNCs (Fig. 1g) indicated that 73.16% of FCFNCs particles were around 66.3 nm with a standard deviation of 14.02%, while the hydrodynamic size of FCNPs, FFNPs was assessed by 83.2 nm (63.5%) and 21 nm (57.4%) with a standard deviation of 17.62% and 19.36%, respectively (Supplementary Fig. S5a and b). Such difference in size between TEM and PSD could be assigned to the presence of water and other biomaterial molecules associated with the surface of NPs^4^.

#### Surface charge characteristics

The electrokinetic potential of the streptogenic NPs and their long-term colloidal stability were evaluated by ζ-potential. It recorded − 31.3 mV, − 36.4 mV and − 26.2 mV for FCNPs and FFNPs and FCFNCs, respectively (Supplementary Fig. S5c and d and Fig. 1h). The stability limit of particles was in the range ± 25 mV, i.e., particles with ζ-potential values more than + 25 mV or less than − 25 mV exhibit higher electrostatic interaction, which provided them stability with a little chance for flocculation and agglomeration^4,43^. Accordingly, both FCNPs and FFNPs displayed higher stability and better monodispersity than FCFNCs, which could be attributed to the electro-static repulsion force among the smaller adjacent NPs that led to Brownian motion; eventually retaining NPs suspension away from aggregation and disposition. Negatively charged surface of the NPs indicated their association with negatively charged biomolecules such as polyacids of protein moieties and the sugar-phosphate backbone of nucleic acid moieties. Notably, ζ-potential of as-prepared nanomaterials in this study showed better results than other related investigations^12,38^.

#### Biosynthesis mechanism

**Figure 2:**
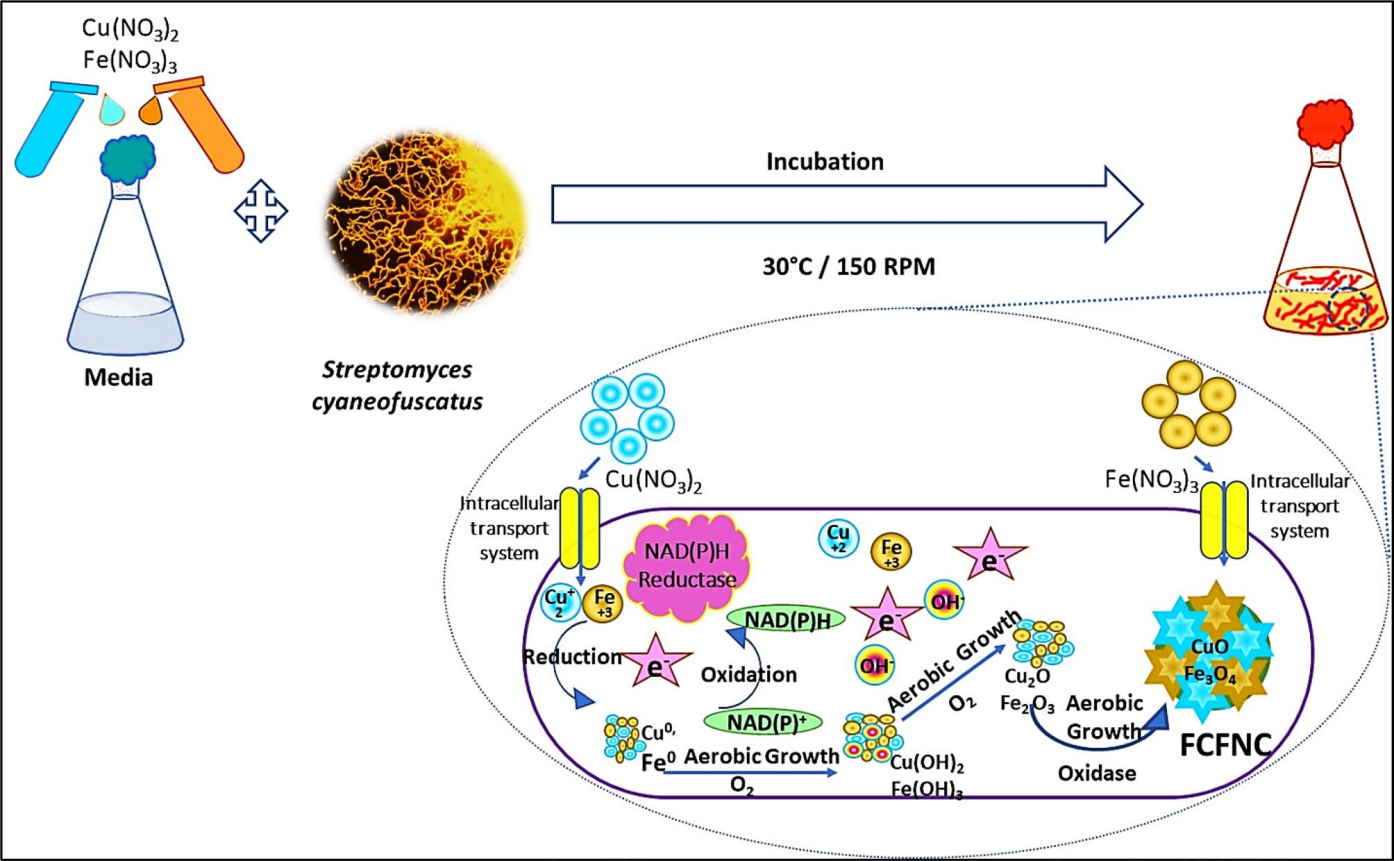
Schematic diagram representing the biosynthesis process of FCFNCs via *S. cyaneofuscatus* EM3 in presence of Cu(NO_3_)_2_ and Fe(NO_3_)_3_.

Based on the preceding experimental data, the following sequence of events could be proposed to explicate the mechanism of NPs synthesis via *S. cyaneofuscatus* EM3. The mechanism involved a cascade of stages that fall in the scope of oxidation–reduction (redox) reaction illustrated in Fig. 2. Initially, the positively charged ions of metal precursors interacted electrostatically with different negatively charged functional groups that characterize actinomycetes cell wall^10^. Through various uptake systems, the acquired metals were internalized into the cell either to mitigate their toxicity or to utilize them in bacterial metabolism. In this sense, it is worth mention that the critical roles of Cu and Fe as the most common redox-active metals that conjugate with proteins^44,45^. The bioreduction step began once the metals entered the cells. Thereafter, the intracellular compartmentalization occurred due to the zero-oxidation Cu/Fe nuclei produced by the process summarized in the following equations

1 $$ {\text{H}}_{2} {\text{O }} \to {\text{ H}}^{ \cdot } + {\text{ OH}}^{ \cdot } $$

2 $$ {\text{Cu}}^{2 + } + \, 2{\text{H}}^{ \cdot } + {\text{ NAD}}({\text{P}}){\text{H-reductase}} \to {\text{ Cu}}^{ \circ } + \, 2{\text{H}}^{ + } $$

3 $$ {\text{Cu}}^{ + } + {\text{ H}}^{ \cdot } + {\text{NAD}}({\text{P}}){\text{H-reductase }} \to {\text{ Cu}}^{ \circ } + {\text{ H}}^{ + } $$

4 $$ {\text{Fe}}^{3 + } + \, 3{\text{H}}^{ \cdot } + {\text{NAD}}({\text{P}}){\text{H-reductase }} \to {\text{ Fe}}^{ \circ } \, + \, 3{\text{H}}^{ + } $$

5 $$ {\text{Fe}}^{2 + } + \, 2{\text{H}}^{ \cdot } + {\text{NAD}}({\text{P}}){\text{H-reductase }} \to {\text{ Fe}}^{ \circ } \, + \, 2{\text{H}}^{ + } $$

Remarkably, various microbial biomolecules could participate substantially in the reduction phase and chelating activity to transform metal ions into their NPs analogues, including NADH-dependent enzymes, such as cytochrome reductase, hydrogenases, nitrate reductase, dehydrogenases^4^, superoxide dismutase and catalase^46^. Actually, in the biological redox reaction, the enzymes shuttle electrons to the metal ions that were reduced to the NPs through sequential variation in their oxidation states. During aerobic growth, the metallic Cu and Fe were oxidized, both by oxygen that was available in aerobic incubation and by the oxidizing biomolecules produced by microbial cells^43^, which generated Cu^2+^, Fe^3+^ and OH^−^ that are responsible for the formation of Cu(OH)_2_, Fe(OH)_2_ and Fe(OH)_3_ on the metallic Cu and Fe, respectively (Eqs. 6, 7 and 8).

6 $$ {\text{ Cu}}_{{({\text{s}})}} + \, 2\;{\text{OH}}^{ - } + {\text{ Oxidase }} \to {\text{ Cu}}\;({\text{OH}})_{{2({\text{s}})}} + \, 2{\text{H}}_{2} {\text{O}} $$

7 $$ {\text{Fe}}_{{({\text{s}})}} + 2\;{\text{OH}}^{ - } + {\text{ Oxidase }} \to {\text{ Fe}}\;({\text{OH}})_{{2({\text{s}})}} + \, 2{\text{H}}_{2} {\text{O}} $$

8 $$ {\text{Fe}}_{{({\text{s}})}} + 3\;{\text{OH}}^{ - } + {\text{ Oxidase }} \to {\text{ Fe}}\;({\text{OH}})_{{3({\text{s}})}} + \, 3{\text{H}}_{2} {\text{O}} $$

Such hydroxide intermediates were dissociated to CuO, FeOOH and Fe_3_O_4_ as manifested in Eqs. (9), (10) and (11) below

9 $$ {\text{Cu}}\;({\text{OH}})_{4}^{2 - } \to {\text{CuO}}_{{({\text{s}})}} + \, 2\;{\text{OH}}^{ - } + {\text{ H}}_{2} {\text{O}} $$

10 $$ {\text{Fe}}\;({\text{OH}})_{{3({\text{s}})}} \to {\text{ FeOOH}}_{{({\text{s}})}} + {\text{ H}}_{2} {\text{O}} $$

11 $$ 2\;{\text{Fe}}^{3 + } + {\text{ Fe}}^{2 + } + \, 8\;{\text{OH}}^{ - } \to \, 2\;{\text{Fe}}\;({\text{OH}})_{3} {\text{Fe}}\;({\text{OH}})_{2} \to {\text{ Fe}}_{3} {\text{O}}_{4} + 4\;{\text{H}}_{2} {\text{O}} $$

Simultaneously, under continuous aerobic incubation and at different microbial growth phases, the development of other oxides (e.g., Cu_2_O and Fe_2_O_3_) was observed as inferred from XRD (Fig. 1a). The reactions were presented in Eqs. (12) and (13).

12 $$ 4\;{\text{Cu }} + {\text{ O}}_{2} \to \, 2\;{\text{Cu}}_{2} {\text{O}} $$

13 $$ 4\;{\text{Fe }} + \, 3\;{\text{O}}_{2} \to \, 2\;{\text{Fe}}_{2} {\text{O}}_{3} $$

However, both products could be further oxidized to CuO and Fe_3_O_4_, which are the most stable phases, according to the following equations

14 $$ 2\;{\text{Cu}}_{2} {\text{O }} + {\text{ O}}_{2} \to \, 4\;{\text{CuO}} $$

15 $$ 3\;{\text{Fe}}_{2} {\text{O}}_{3} + \, 1/2\;{\text{O}}_{2} \to \, 2\;{\text{Fe}}_{3} {\text{O}}_{4} $$

Interestingly, in the biosynthesis of FCFNC, the simultaneous redox of both metals was probably followed by *S. cyaneofuscatus* EM3, which implied cellular utilization of both metals at the same time without ionic selectivity property. This gave rise to such nanocomposite architecture (CuO/Cu_2_O/Cu/Fe_3_O_4_/FeOOH). In fact, the assembly of any binary elements to different composite nanoforms is based on several parameters that include the precursors ratio, pH, energy barrier, atomic size, oxidation/reduction potentials, electronegativity, and electronic/magnetic effects as well^9,16^. For FCFNCs in the current study, despite there being a difference in reduction potential of Fe^3+^/Fe^0^ (E^0^ = − 0.44 V) and Cu^2+^/Cu^0^ (E^0^ = 0.34 V)^16^, the nanocomposite with Cu and Fe in equivalent proportion was generated, as revealed by EDX. That could be attributed to the influence of pH. During *S. cyaneofuscatus* EM3 growth phases, pH fluctuated between 6.7–8.0 as a consequence of nitrate utilization and its conversion into nitrite (NO_2_^−^) and ammonia (NH_3_^+^) by the act of nitrate reductase enzyme. Under these circumstances, Fe was rapidly reduced at a higher rate than Cu, which favors acidic conditions for precipitation^32,47^; forming Fe_3_O_4_ without any existence of Fe and/or Fe_2_O_3_ in the hybrid system (Fig. 1a). Nevertheless, the slower rate of Cu reduction led to the predominance of CuO on the surface of Cu_2_O and Cu, which existed in relatively low concentration. Therefore, in this study, the mutual effect of both redox potential and pH managed FCFNC synthesis in such configuration. These deductions were commensurate well with the earlier findings^32,47^.

### Applications of as-prepared NPs

#### Cytotoxicity and anticancer activity of as-prepared NPs

Cancer is the second leading cause of human death. It is estimated that about 9.6 million deaths were caused by cancer in 2018 across the world^48^. The current form of chemotherapy did not cause a significant reduction in mortality among cancer patients; thus, the new and safer approaches are critically needed. Nanotherapy is the rational alternative remedial approach. The initial studies used NPs as drug carriers in which anticancer drugs were entrapped. Thereafter, the intrinsic antitumor effect of NPs was discovered, and their use as a drug by themselves to conquer tumor development began^49^. The potential of metallic NPs, especially transition metals (e.g., copper and iron) in the role of tumor inhibitors due to their unique properties and low toxicity has been demonstrated.

**Figure 3:**
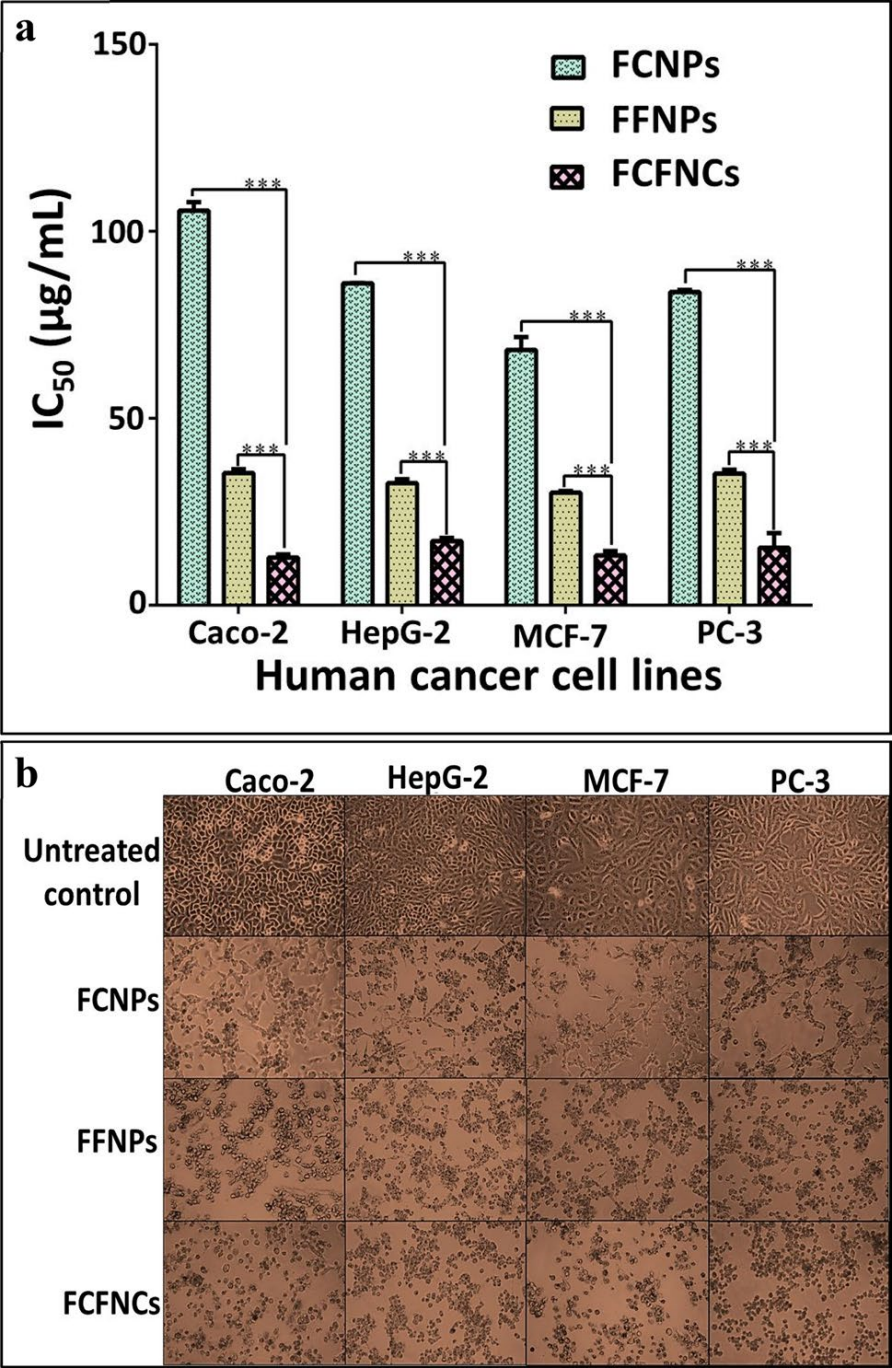
MTT results of the tested NPs against human cancer cells. IC_50_ (μg/mL) values (**a**) with the morphological alterations of cancer cell lines after 72 h treatment with these tested NPs (**b**). All values were expressed as mean ± SEM. FCFNCs were compared with FCNPs and FFNPs. This comparison considers significantly different at *p < 0.05**,** **p < 0.005, ***p < 0.0005.

As recorded formerly, Cu and Fe are functional elements for many cellular processes, yet, in high concentrations, they adversely affect^50^. Therefore, herein, we examined the impact of all biosynthesized NPs on the viability of normal human cells. The safe doses (EC_100_) of FCNPs, FFNPs and FCFNCs that maintained 100% viability of normal human lung fibroblast Wi-38 cells were 823.5 ± 6.2 μg/mL, 1258.6 ± 24.8 μg/mL and 1999.7 ± 21.5 μg/mL, respectively. The lowest IC_50_ level of IC_50_ ≤ 17.21 μg/mL that had the strongest potential anticancer effect on all studied human cancer cells was demonstrated by hybrid FCFNCs. Figure 3a presents high anticancer activity of FFNPs (IC_50_ 30.10–35.36 μg/mL) against all tested human cancer cell lines in comparison to FCNPs (IC_50_ 83.77–105.52 μg/mL). Further, IC_50_ values of metal oxide NPs in the treated cancer cells were lower than their EC_100_ values in the treated normal cells. This reflected the selective preference of these metal oxide NPs for cancer cells, which has been reported in a recent study^51^. Previous studies found that CuNPs synthesized by *Bacillus cereus* display a similar effect at IC_50_ ≥ 20 μg/mL on MCF-7, Caco-2 and HepG2 cells^52^, while CuNPs produced by *Sargassum polycystum* brown seaweed at IC_50_ = 61.25 μg/mL were effective on MCF-7^53^. On the other hand, Namvar et al.^54^ recorded the effect of IC_50_ of 18.75 μg/mL and 23.83 μg/mL of iron oxide (IO) NPs synthesized by *Sargassum muticum* (seaweed) on MCF-7 and HepG2 cells, respectively.

Moreover, the morphological changes of FCFNCs-treated cells, including the complete collapse of the normal spindle shape and cell shrinkage, proved the strongest anticancer effect of FCFNCs among all examined NPs (Fig. 3b). More importantly, the combination index (CI) of FCFNCs anticancer activity was estimated at 50% inhibition in order to characterize the type of interaction between FCNPs and FFNPs as synergism, additivity or antagonism (< 1, = 1 or > 1, respectively). Therefore, the powerful anticancer effect of the hybrid nanocomposite might be attributed to high synergism between CuNPs and FeNPs (CIs = 0.422 ± 0.014, 0.628 ± 0.045, 0.543 ± 0.059 and 0.526 ± 0.122 against Caco-2, HepG-2, MCF-7 and PC-3, respectively). However, to our knowledge, the detailed synergistic antitumor features of FCFNCs were not studied before. Hence, further analysis of apoptotic mechanism using flow cytometry, fluorescence microscopy, immunostaining Ki-67 and expression levels of tumor suppressor gene (p53) and caspase 3 were investigated here.

##### Apoptosis-induced anticancer effect of as-prepared NPs

**Figure 4:**
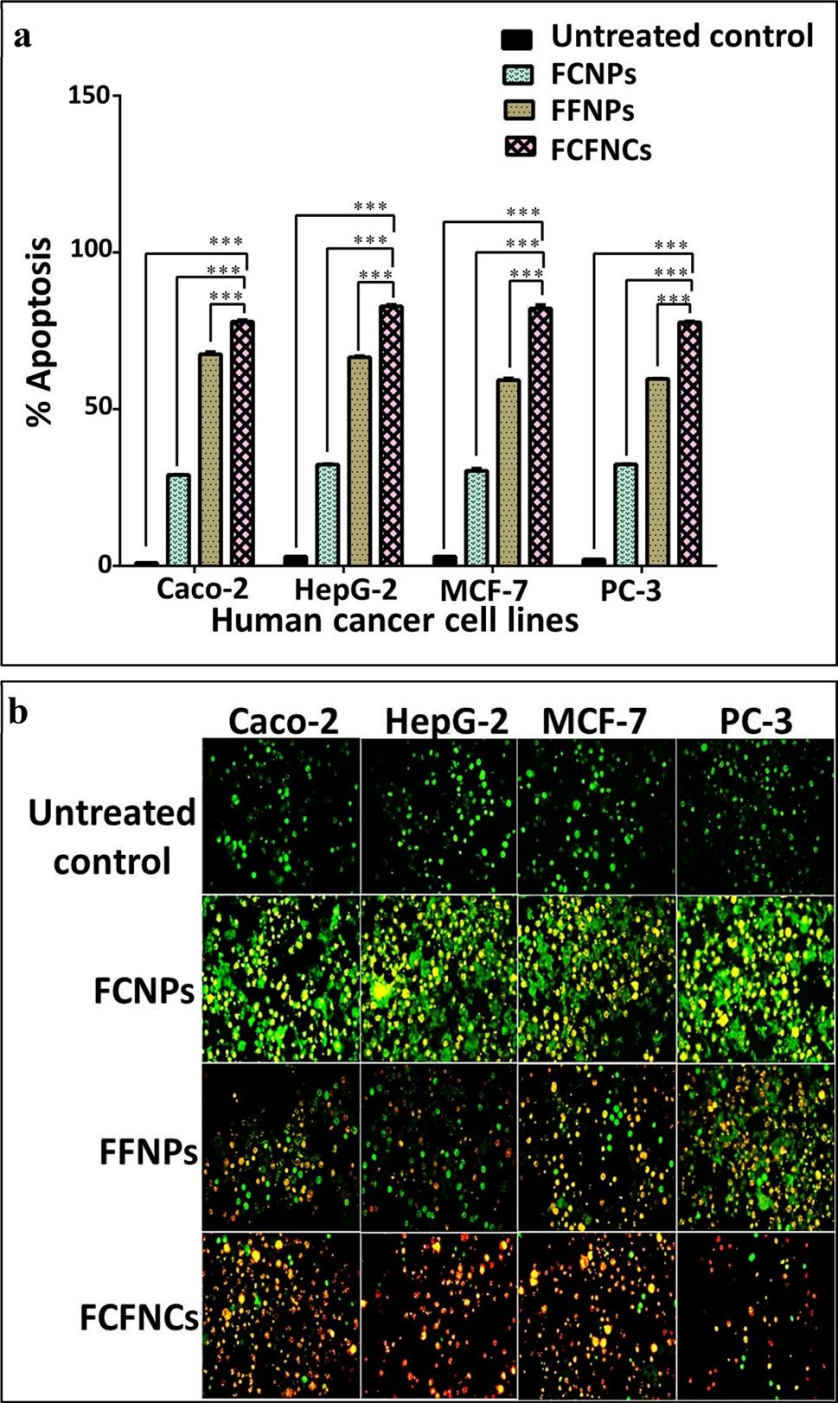
Apoptotic cell population in FCNPs*,* FFNPs and FCFNCs-treated human cancer cells compared with the untreated cancer cell lines. The percentage of apoptosis in the control and treated cancer cells using flow cytometry after staining with annexin V/propidium iodide (**a**). Fluorescence images of AO/EB double staining with green, yellowish-orange, and red fluorescence indicate viable, early apoptotic and late apoptotic cells, respectively (magnification × 200) (**b**). All values were expressed as mean ± SEM. FCFNCs were compared with FCNPs and FFNPs. This comparison considers significantly different at *p < 0.05, **p < 0.005, ***p < 0.0005.

In Fig. 4a and Supplementary Fig. S6, the apoptotic potential of FCNPs, FFNPs and FCFNCs was illustrated using flow cytometric analysis by higher percentages of annexin V-stained apoptotic cells than that of propidium iodide-stained necrotic cancer cells. The percentages of annexin V-stained apoptotic cell populations in FCNPs-treated cancer cell lines were < 33%. In comparison, 59.27–67.56% apoptosis was estimated in the case of FFNPs treated cancer cells. Remarkably, FCFNCs exhibited the highest apoptosis percentage (77.71–82.8%) for all tested human cancer cells. Remarkably, a synergistic apoptotic anticancer effect (CI 0.619 ± 0.008, 0.558 ± 0.003, 0.585 ± 0.006 and 0.592 ± 0.009) was recorded between CuNPs and FeNPs in FCFNCs-treated colon, liver, breast and prostate cancer cells, respectively. Furthermore, the apoptosis was clarified with acridine orange/ethidium bromide (AO/EB) staining and visualized using a fluorescence microscope (Fig. 4b). The NPs-treated cancer cells had fluorescence ranging from yellow to red in comparison with the control (untreated viable cancer cells) that showed green fluorescence. The low anticancer effect of FCNPs was confirmed by yellow fluorescence staining of the treated cancer cells. While the nuclei of FFNPs-treated cancer cells showed orange fluorescence, which indicated high apoptotic effect. Notably, the most potent anticancer effect of FCFNCs was illustrated by red fluorescence of the nuclei of the treated colon, liver, breast and prostate cancer cell lines.

##### Prooxidant effect-mediated anticancer activity of as-prepared NPs

**Figure 5:**
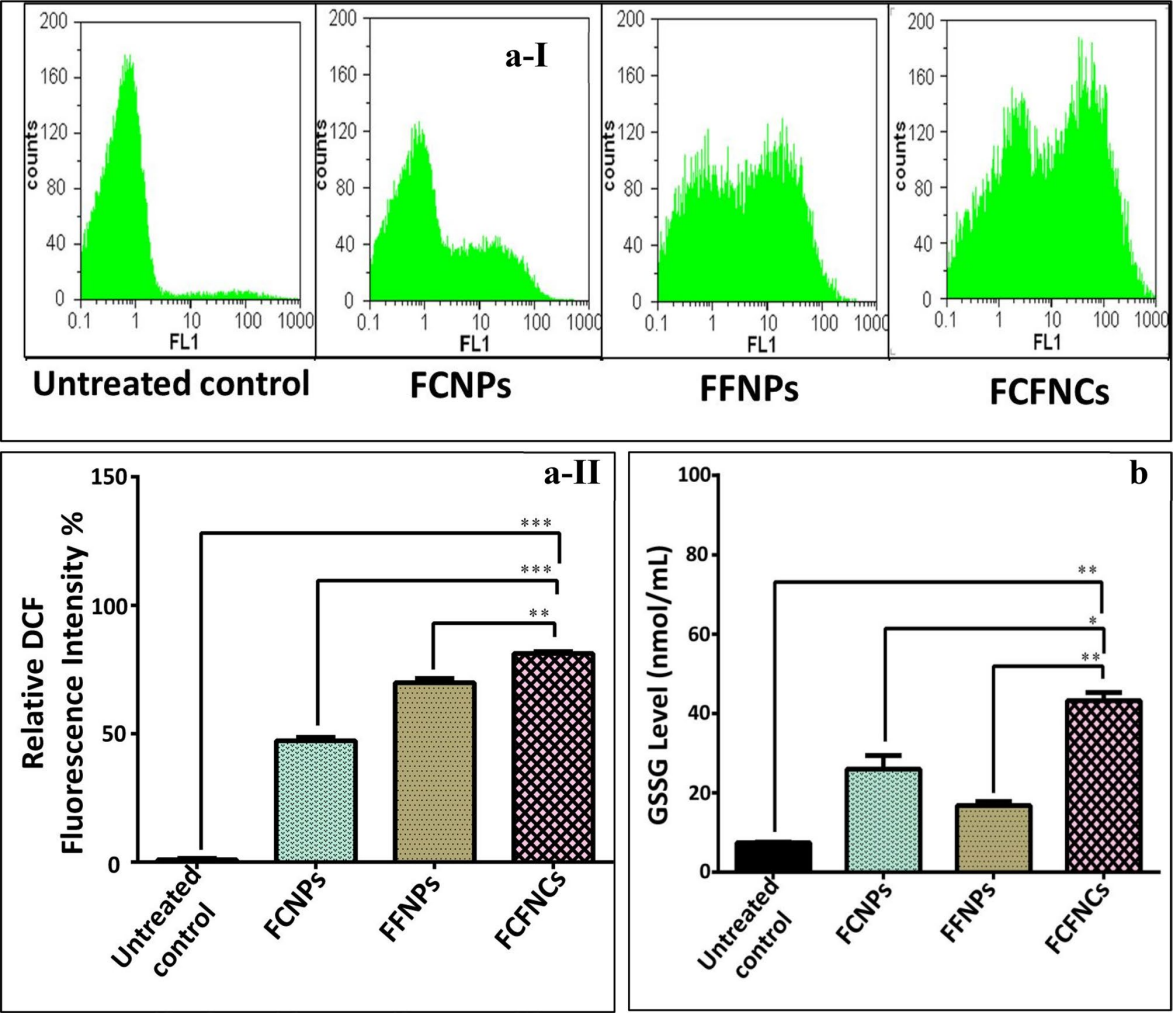
Prooxidant effect of FCNPs, FFNPs and FCFNCs in the treated HepG-2 cells relative to the untreated cancer cell lines. (**aI**, **II**) Flow cytometric charts of fluorescence oxidized form of dichlorofluorescin diacetate (indicator of reactive oxygen species “ROS”) with quantitative histogram for the percentage of intracellular ROS. (**b**) Cellular content of GSSG (nmol/mL) in FCNPs, FFNPs and FFNPs -treated HepG-2 relative to the untreated cells. All values were expressed as mean ± SEM. FCFNCs were compared with all other treatments with significance at *p < 0.05, **p < 0.005, ***p < 0.0005.

One promising approach for collapsing cancer cells is enhancing the lethal reactive oxygen species (ROS) generation^55^. So, the redox activity of the active NPs was detected in HepG-2 cells (the most sensitive cancer cell line to be the tested with NPs based on previously mentioned results) using the ROS-sensitive fluorescent probe (DCF). As shown in Fig. 5a-I, II, the incubation of HepG-2 cells with FCNPs, FFNPs and FCFNCs caused a dramatic increase in the ROS level by 47.305 ± 1.34%, 69.925 ± 1.62% and 81.29 ± 0.75%, respectively. Also, GSSG level increased in FCNPs, FFNPs and FCFNCs-treated HepG-2 cells (26.58 ± 3.4, 16.81 ± 1.54 and 43.24 ± 2.01 nmol/mL, respectively) compared to 7.39 ± 0.17 nmol/mL in the untreated HepG-2 cells (Fig. 5b). This indicated that FCFNCs-treated HepG-2 cells had the highest levels of ROS and GSSG. Moreover, a synergistic interaction (CI = 0.721 ± 0.025 and 0.500 ± 0.034) between the prooxidant effect of CuNPs and FeNPs was recorded for ROS and GSSG, respectively in hybrid FCFNPs-treated HepG-2 cells. Therefore, our hybrid nanocomposites of Cu and Fe which demonstrated the highest prooxidant effect, were the most powerful inducer of the apoptosis-dependent death in all studied human cancer cell lines. Interestingly, Plotek et al.^56^ and Jungwirth et al.^57^ highlighted that Cu and Fe exhibited prooxidant-dependent nuclease activity by enhancing the formation of ROS, particularly hydroxyl radical which is the main cause of DNA damage mediate cell death. Dixon and Stockwell^55^ referred to transition metals (particularly, iron) and ROS as the most important triggers for cell death mediation. The synergistic prooxidant effect dependent anticancer activity of FCFNCs might be attributed to copper (II) which potentiates the prooxidant effect of Fe (III) by reducing it to Fe (II) by ROS produced by Cu (II)-catalyzing the oxidation of glutathione. This is elucidated by higher GSSG level in the FCNPs-treated HepG-2 cells than FFNPs-treated HepG-2 cells as demonstrated by the current study. To sustain their uncontrolled proliferation, cancer cells promote the reduction of GSSG to maintain a highly reduced microenvironment which prevent the accumulation of lethal ROS^55,58^. In conclusion, the strongest synergistic anticancer effect of hybrid NPs may due to their highest synergistic prooxidant activity that aggravated oxidative damage and enhanced the multiplicative apoptosis-dependent death among the FCFNCs-treated cancer cells.

##### The gene expression levels of tumor suppressor gene (p53) and caspase 3 and immunohistochemical expression of proliferation marker (Ki-67) in the NPs-treated cancer cells

**Figure 6:**
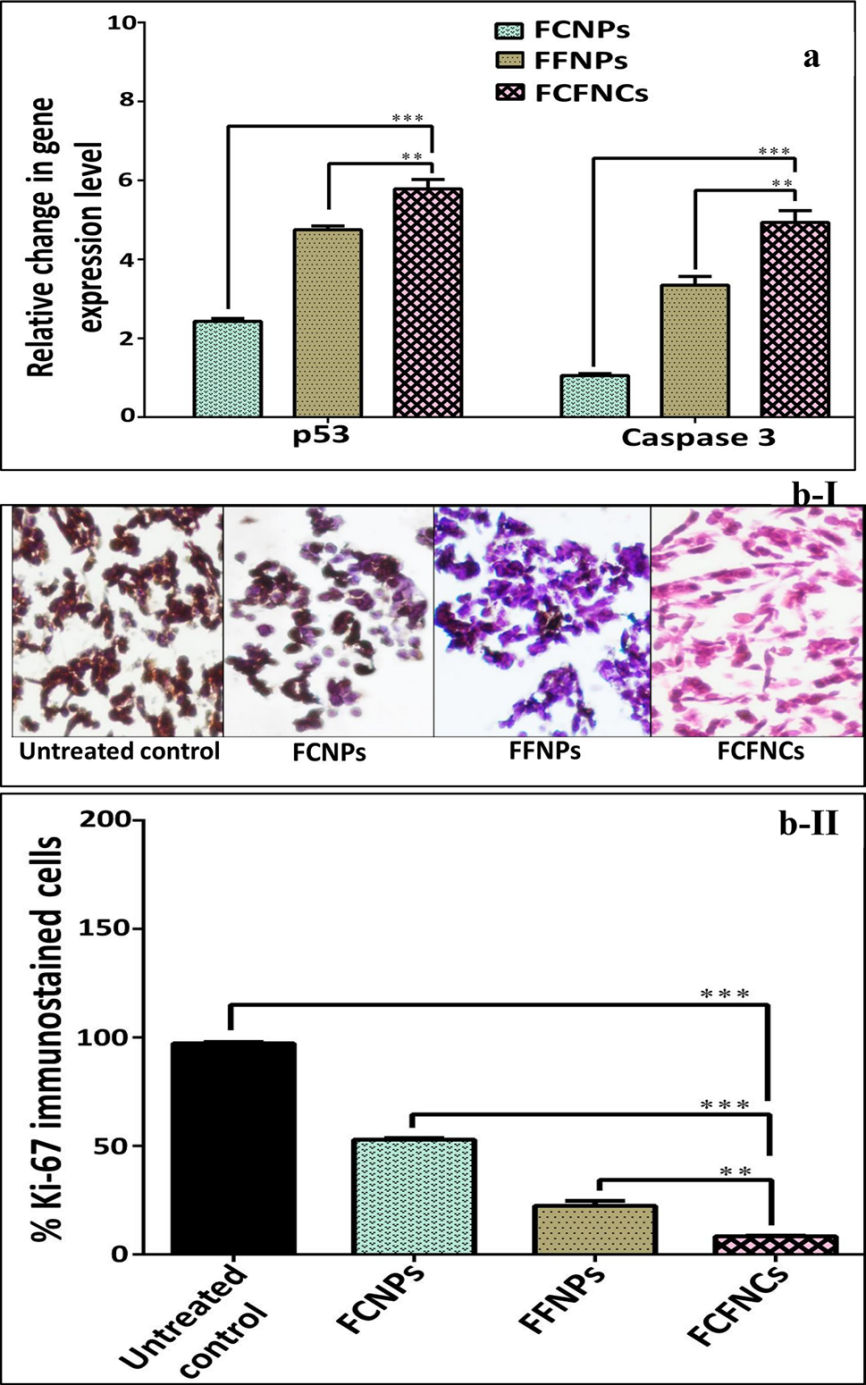
Impact of FCNPs*,* FFNPs and FCFNCs on apoptotic and proliferation markers in the treated HepG-2 cells relative to the untreated cancer cell lines. (**a**) Relative change in the gene expression of p53 and caspase 3 using QPCR. (**b-I**, **II**) Microscopic image for Ki-67 of the untreated and treated cancer cells (magnification × 400) with representative percentage of positive immunostaining cancer cells. All values were expressed as mean ± SEM. FCFNCs were compared with all other treatments with significance at *p < 0.05, **p < 0.005, ***p < 0.0005.

Notably, FFNPs were able to upregulate the expression of p53 and caspase 3 (apoptosis executors) by 4.7 and 3.3 folds, respectively, while FCNPs did not show any marked change in the expressions of caspase 3. A change was recorded only in the expression of p53 as an increase by 2.4 folds in the treated HepG-2 cells (Fig. 6a). However, the hybrid nanocomposite was able to increase the expression of p53 and caspase 3 about 5 folds in the treated HepG-2 cells. Accordingly, it was found that CIs of FCFNCs for up-regulation p53 and caspase 3 expressions were 0.801 ± 0.15 and 0.621 ± 0.011, respectively, indicating the synergistic interaction. Noteworthy mention that p53 is one of the oxidative stress response transcription factors^59^. Thus, only up on ROS activation by these transition metals does; the apoptosis induction occurs. Maqusood et al.^60^ illustrated the anticancer effect of IONPs on HepG2 and A549 (human lung adenocarcinoma) cells via inducing ROS as well as p53, caspase 3 and caspase 9 expressions that trigger apoptosis. Our findings agreed with Shafagh et al.^61^ who showed that the generation of ROS, up-regulation of p53 expression and unchanged caspase 3 expression in CuO NPs-treated K562 leukemia.

Further, FFNPs-treated cancer cells revealed 22.47% Ki-67 staining (a marker for cell proliferation), which was lower than that (52.94%) in FCNPs-treated cancer cells (Fig. 6bI, II). Obviously, FCFNCs-treated cancer cells demonstrated the lowest percentage (8.3%) of Ki-67 staining (Fig. 6bI, II) with a CI value equals to 0.222, which considered as a sign of synergism between CuNPs and FeNPs in biosynthesized hybrid nanocomposites. Consequently, the utilization of biosynthesized FCFNCs in nanotherapy deemed promising by the dint of the enhanced properties of such hybrid that enable halting tumor progression.

#### Antimicrobial activity

The commercial and traditional organic antimicrobial agents suffer from several shortcomings such as toxicity to human beings, environmental pollution and increasing the chance of the microbes developing multidrug-resistance. Consequently, the recent development of inorganic agents is critical, especially the metals NPs, which exhibiting customized features of antimicrobial functionalities, without contaminating the ambient environment and avoiding damage the tissue in in-vivo applications like wound-healing. There are multidisciplinary applications of such biocidal metal-NPs in food packaging, water disinfection, dental restorative, medical devices, bone prostheses, surgical instruments, smart textiles and skin care products^62^. Accordingly, the antimicrobial, antibiofilm, algicidal activity of FCFNCs were compared to their sole NPs and antibiotics, at concentrations (250, 500 and 750 µg mL^−1^).

The cell viability was assessed using the turbidity assay. The data presented in Table 2 indicated that the reduction in the microbial viability induced by all the evaluated nano-species occurred in a dose-dependent manner. Also, the FCFNCs exhibited superior suppression activity than that displayed by their sole counterparts. The statistical analysis of the data with one-way ANOVA showed that treatments with NPs were significantly effective (*P ˂ *0.05). Further, the maximum inhibition percentages, 92.7 and 81.6% were observed against *B. cereus* and *S. aureuse*, respectively, at 500 µg mL^−1^ of FCFNCs. These results were similar to those reported by Kim et al.^63^, and comparable relatively to those obtained by Bakina et al.^12^. Notably, *B. cereus* was most susceptible to all examined antimicrobial agents with all concentrations, while *P. aeruginosa* showed comparatively greater resistance. Hence, the image data demonstrates the sensitivity order of microbial pathogens versus examined NPs, which could be summarized as *B. cereus ˃ S. aureus ˃ E. faecalis ˃ E. coli ˃ S. typhi ˃ P. aeruginosa.* Moreover, 48.0 ± 0.81 and 22.5 ± 0.57 of viability percentages were noticed respectively for *C. albicans* under the stress of hybrid nanocomposite at concentrations 250 and 500 µg mL^−1^. This reflects the significant capability of the NPs to penetrate and corrupt the robust glycoprotein-glucan-chitin structure of fungal cell wall^2,4^. Though tetracycline and nystatin (reference antibiotics) were more effective, FCFNCs may be on a par with them in the term of activity and thereby could compete economically, especially at higher concentration. In contrast, Akinsiku et al.^41^ recorded lower efficiency of AgNi bimetallic nanostructures in comparison with the conventional antibiotics, despite well-known toxicity of silver and nickel. Ultimately, all tested planktonic pathogens were almost completely inhibited by FCFNCs with a dose of 750 µg mL^−1^.

**Table 2:** The antagonistic efficiency of FCNPs, FFNPs, FCFNCs and antibiotics (tetracycline for bacteria and nystatin for yeast) at 250, 500 and 750 µg mL^−1^ against planktonic prokaryotic and eukaryotic pathogens.

Concentrations (µg mL−^1^)	Treatments	Inhibition %						
		*E. coli*	*S. typhi*	*P. aeruginosa*	*S. aureus*	*B. cereus*	*E. faecalis*	*C. albicans*
250	FCNPs	24.0 ± 0.11**	22.5 ± 0.28**	21.0 ± 0.12**	31.2 ± 0.15**	35.8 ± 0.07**	30.3 ± 0.17**	27.0 ± 0.07**
	FFNPs	28.2 ± 0.19**	25.0 ± 0.06**	19.2 ± 0.17**	35.3 ± 0.17**	39.3 ± 0.32**	31.5 ± 0.3**	31.8 ± 0.03**
	FCFNCs	47.6 ± 0.4	45.5 ± 2.5	37.4 ± 1.05	59.79 ± 0.29	64.4 ± 0.315	57.3 ± 0.56	52.1 ± 0.19
	Antibiotic	80.8 ± 0.35**	79.3 ± 0.30**	74.0 ± 0.025**	81.4 ± 0.29**	88.7 ± 0.40**	83.5 ± 0.40**	75.4 ± 0.30**
500	FCNPs	52.2 ± 0.28**	51.2 ± 0.25**	46.0 ± 0.19**	58.4 ± 0.36**	61.8 ± 0.33**	54.4 ± 0.35**	53.0 ± 0.16**
	FFNPs	58.5 ± 0.41**	54.5 ± 0.7**	41.5 ± 0.32**	61.5 ± 0.30**	67.2 ± 0.17**	60.4 ± 0.37**	57.0 ± 0.26**
	FCFNCs	78.3 ± 0.56	75.0 ± 0.05	70.8 ± 0.10	81.6 ± 0.61	92.7 ± 0.14	80.6 ± 0.54	77.5 ± 0.43
	Antibiotic	98.4 ± 0.4**	95.2 ± 0.2**	94.2 ± 0.3**	95.5 ± 0.21**	99.4 ± 0.1**	99.2 ± 0.15**	91.1 ± 0.4**
750	FCNPs	80.5 ± 0.4**	75.9 ± 0.8**	70.7 ± 1.5**	90.8 ± 0.5**	96.7 ± 1.0**	88.1 ± 0.5**	80 ± 0.9**
	FFNPs	85.6 ± 1.1**	81.4 ± 1.0**	78.4 ± 0.5**	95.7 ± 0.9**	98.5 ± 0.9**	91.6 ± 0.7**	87.2 ± 0.8**
	FCFNCs	95.8 ± 1.2**	92.5 ± 1.2**	88.7 ± 1.4**	100 ± 0.0**	100 ± 0.0**	98.8 ± 0.9***	97.5 ± 0.7***
	Antibiotic	100 ± 0.0***	100 ± 0.0***	100 ± 0.0***	100 ± 0.0***	100 ± 0.0***	100 ± 0.0***	100 ± 0.0***

All values were expressed as mean ± SEM. FCFNCs were compared with all other treatments, at each concentration, with significance at *p < 0.05, **p < 0.005, ***p < 0.0005.

#### Antibiofilm activities (synthesis and detachment)

A biofilm lifestyle adopted by planktonic cells that encapsulated themselves in a self-produced protective polymeric matrix, which thereafter adhered to biotic and abiotic surfaces whether in natural, clinical, or even industrial and food-processing environments^43^. By such type of lifestyle, the microbial cells are refractory to disinfectants, antibiotics and host’ immune systems, which lead to outbreaks and environmental/industrial fouling issues^2^. Hence, the application of NPs in biofilm eradication during biofilm formation or disintegration of the preformed one is an important solution. In the current study, noticeably progressive inactivation of biofilm formation that increased with increasing concentrations of all treatments was observed (Table 3). Analysis of variance showed that FCFNCs at 750 µg mL^−1^ caused significant inhibition (*P *˂ 0.05) of biofilm growth in *S. aureus* (90.6 ± 1.3%) and *P. aeruginosa* (76.11 ± 1.17%). In comparison, Chatzimitakos and coworkers^46^ found that 100 mg/mL of CuFe nanohybrid corrupted the biofilm formation of *E. coli* and *S. aureus* by 12 and 49%, respectively, proofing the efficiency of our FCFNCs in minuscule amounts.

**Table 3:** Antibiofilm potency of FCNPs, FFNPs, FCFNCs and the antibiotic (tetracycline) at different concentrations in response to biofilm evolution and degradation.

Concentrations (µg mL^−1^)	Treatments	Inhibition %				
		Biofilm formation		Biofilm disintegration		*C. vulgaris*
		*S. aureus*	*P. aeruginosa*	*S. aureus*	*P. aeruginosa*	
500	FCNPs	39.84 ± 1.5	29.76 ± 1.4	25.18 ± 2.8**	14.64 ± 1.4*	29.39 ± 0.39***
	FFNPs	49.72 ± 1.2	27.03 ± 1.2	19.33 ± 1.4**	10.4 ± 0.51*	29.94 ± 1.9*
	FCFNCs	77.42 ± 2.7	55.48 ± 1.1	31.2 ± 1.2	23.28 ± 1.14	42.36 ± 3.7*
	Tetracycline	79.97 ± 0.7	83.9 ± 3.09	68.13 ± 2.1**	61 ± 0.7**	80.20 ± 4.4*
750	FCNPs	54.75 ± 0.3	42.58 ± 1.4	39.9 ± 1.1**	28.82 ± 1.8**	48.96 ± 2.81*
	FFNPs	60.98 ± 0.29	41.19 ± 1.1	40.54 ± 0.6**	21.42 ± 0.92**	60.63 ± 2.2*
	FCFNCs	90.6 ± 1.3*	76.78 ± 0.28	58.87 ± 0.7*	41.81 ± 0.76*	79.31 ± 0.88*
	Tetracycline	93.47 ± 1.0	90.97 ± 1.38	72.27 ± 1.3**	64 ± 2.19**	84.57 ± 1.2***

All values were expressed as mean ± SEM. FCFNCs were compared with all other treatments, at each concentration, with significance at *p < 0.05, **p < 0.005, ***p < 0.0005.

Meanwhile, the biofilm that are already formed showed notoriously tolerance to all treatments. Nonetheless, treatment with 750 µg mL^−1^ of FCFNCs induced the highest significant effective detachment of *S. aureus* and *P. aeruginosa* bulk biofilms reached 58.8 ± 0.8 and 41.8 ± 0.8%, respectively. Such lessness in the antibiofilm efficiency exerted against preformed biofilm deemed intuitive and expected due to the difficulties arose from the contact and interaction between NPs and bacterial cells that are colonized in robust, low permeable and pervasive extracellular polymeric substance (EPS). Let alone higher resistance of stationary cells in the dormant zone of biofilms. As documented by Xiong^62^, the influence of antimicrobial agents is constrained against dormant microbial cells. Therefore, it is plausible to mention that the higher antibiofilm potency of NPs during growth and evolution of biofilm could be ascribed to the vulnerability of the cells at the planktonic or the early stages of colonization, the direct and better contact between NPs and cells before the formation of polymeric barrier and also the possibility of destruction quorum sensing system^2^. Out of these results, the efficacy of FCFNCs of this study in the abatement of biofilm formation and disintegration of its architecture encourage their application as an antibiofilm agent in medical devices and also in fouling prevention.

#### Algicidal activity

The exposure of *C. vulgaris* cells to biofabricated FCNPs and FFNPs and FCFNCs decreased the cell viability significantly in all examined concentrations as revealed statistically (Table 3). Remarkably, the maximum suppression percentage exerted by FCFNCs of 500 and 750 µg mL^−1^ concentrations were 42.36 ± 3.7 and 79.31 ± 0.88%, respectively. The NPs may be ranked as follows, in the order of toxicity strength against *C. vulgaris*, FCFNCs > FFNPs > FCNPs. This considerable algicidal efficacy of FCFNCs empowers their employing in water and wastewater treatment systems to control the environmental issues of algal blooms propagation such as eutrophication and biofouling.

Notably, in this study, FCNPs and FFNPs displayed a considerable significant toxicity. On contrary, Tabrizian^32^ found the chemically prepared iron oxide NPs (Fe_3_O_4_, Fe_2_O_3_) didn’t show any microbial toxicity. Moreover, Liu et al.^64^ recorded a two-fold amelioration in the secondary metabolites production of *S. coelicolor*-M145 by exposure to 10 mg/L of CuO NPs. Such discrepancy in the results in antimicrobial activities among different studies could be attributed to three major parameters. The first one is concerned with NPs, including particle characteristics (type, size, charge, structure, shape, crystal quality, surface area, stability, agglomeration, magnetization, etc.), particle dosage and synthesis method as well^65^. The second reason is the microbial cell concentration and microbial cell characteristics (physiologically and biochemically) which definitely differ not only among various microbial genera, but also among the related species^40,47,66^. The third parameter was the nature of the inactivation process which included the properties of application conditions and the medium in which NPs were applied, including, pH, contact time and organic/inorganic nutrients content of the medium as explained by Pinto et al.^13^ and Noman et al.^36^. Various combinations of all such factors could accelerate or delay the microbial inactivation process and vary its effectiveness.

Broadly, CuFe in its composite nanoform manifested new functionalities and remarkable magnitude of toxicity as compared with FCNPs and FFNPs. That could be assigned to the synergistic effect of two different metals rather than merely additive effect. Other similar studies boosted this point of view^10,14,40,67^. In addition, the driving force of metal- NPs antimicrobial property is the metal ions released from the NPs^62,68^. In this regard, when microbial cells are exposed to different metal ions released by the nanomaterial of a binary nanostructure, the microbe may be suddenly shocked and become unable to develop multiple gene mutations to resist such concurrent, multiple and condensed dose. This may have enabled FCFNCs to target microorganism at numerous sites simultaneously and exerting more than a microbicidal mode of action. Likewise, Noman et al.^36^ discussed similar finding.

Interestingly, iron- and copper-containing nanoparticles (Fe, Fe_2_O_3_, Fe_3_O_4_, Cu, CuO) mostly displayed their toxicity via sequential stages. We could envisage them as nanoknives initiate their action by cracking in the microbial cell wall^4^, diminishing membrane integrity and generating osmotic imbalance^14^. Meanwhile, microbial cells internalize the NPs by endocytotic mechanisms^62^. At this moment, the increased release of metal ions occurs as NPs dissolve intracellularly in a process called Trojan horse mechanism. This causes massive oxidative stress. Then, redox-active particles generate oxygen free radicals or reactive ROS, such as hydroxyl radicals OH^−^, superoxide radicals (O_2_^−^) and singlet oxygen (^1^O_2_), due to the conversion of H_2_O_2_ through Fenton and Haber–Weiss reactions^68,69^.

Actually, elevating the level of ROS in the cell induces programmed death via consecutive paths including, polysaccharides depolymerization, lipid peroxidation, disruption of [4Fe–4S] clusters/thiol-groups of proteins and prohibits DNA replication. Nevertheless, Xiong^62^, and Palza^68^, recommended that the toxicity of metal-NPs could be originated from the replacement of other metal ions in their active sites of biomolecules, in a process called ionic mimicry or molecular mimicry. Aside from that, the metal ions released from metal-NPs interact selectively and/or specifically with some atoms such as oxygen, nitrogen and sulfur in the microbial functional groups; forming complexes that lead to serious alterations in the biomolecules conformational structure which leads eventually to malfunction.

#### Dye detoxification

Azo and triphenylmethane dyes are the most common classes of commercially produced synthetic colorants. For many decades, both types have been utilized as antiseptic agents, fungicides, anthelminthics, ectoparasiticides in aquaculture and commercial fisheries. Also, both recorded as coloring agents and additives in the food processing industry^70^. As well as, they have been used in the biological laboratories as stains, indicators of acidic media and in amyloidosis diagnosing^71^. Remarkably, as effluents, they pose a serious threat to the public health and aquatic ecosystem^72^. Their recalcitrance to degradation hinders light penetration, influences the photochemical activities such as photosynthesis and respiration of aquatic flora/fauna, which subsequently lead to increased chemical oxygen demand (COD) and biological oxygen demand (BOD) and ultimately deteriorate the aquatic ecosystem. Besides, their accumulation in aquatic creatures like fish, mollusks or crustaceans could cause teratogenicity, carcinogenesis, genotoxicity, mutagenicity, hematotoxicity, neurotoxicity as well as multi-organ tissue injuries to human through the food chain^73^.

**Figure 7:**
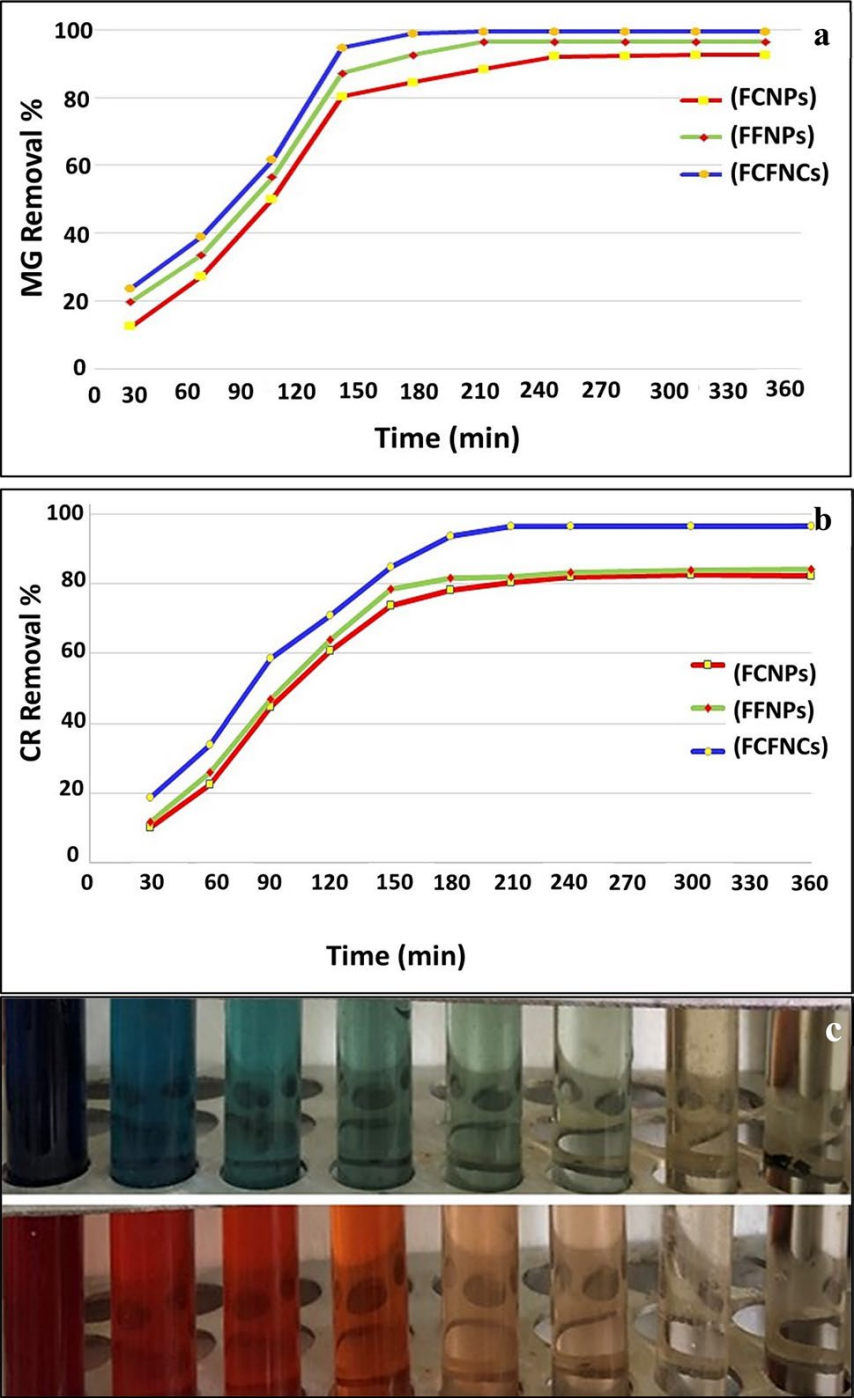
Contact time effect on removal of MG (**a**) and CR (**b**) using FCNPs, FFNPs and FCFNCs. Dye concentration (50 mg/L), NPs dose 1 g/L, temperature (30 °C), pH 7 and agitation speed (150 rpm); (**c**), photographic image showing MG and CR removal by FCFNCs with obvious color fading.

Therefore, the efficacy of FCNPs and FFNPs and FCFNCs was examined for decolorization and detoxification of Congo red (CR) and Malachite green (MG) as a model reaction for anionic and cationic synthetic dyes, correspondingly. Thereafter, the microtoxicity and cytotoxicity of NPs-treated solutions were assessed. To evaluate the appropriate contact time, about 1 g/L of biosorbents FCNPs*,* FFNPs and FCFNCs were agitated with (50 mg/L) CR and MG solutions at different time intervals (30–360 min). As illustrated in Fig. 7, the overall removal process was time-dependent as indicated by gradual fading in the solution color and concurrent decrease in the intensity of absorption band, with time, at 497 nm and 617 nm for CR and MG, respectively. Faster removal was observed in MG solution, reaching up to 61.5 ± 1.5% by FCFNCs within 90 min. After 120 min, there was obvious increment in the removal process reached 94.6 ± 1.1%; a slight difference was shown after 180 min by 99.35 ± 1.8% removal. Concerning CR, only 48.4 ± 2.1% removal was achieved in the first 90 min and reached 93.7 ± 0.5% at 180 min, which remained constant up to 360 min. Conclusively, we could state that 180 min was enough time to obtain a maximum removal of MG and CR. Thus, 180 min as a contact time was selected to explore the effect of different dye concentrations on the removal process.

The initial concentration of MG and CR was varied from 200 to 500 mg/L with constant adsorbents dose (1 g/L); the decolorization percentages are represented graphically in Supplementary Fig. S7. As shown, the removal efficiency of FCFNCs declined from 81.35 ± 0.9 to 57.88 ± 1.5% by increasing MG concentration from 200 to 500 mg/L. Whereas, CR decolorization percentage recorded 68.6 ± 0.7% and 21.49 ± 1.8% at both dosages. In general, the maximum removal percentages were observed at low MG and CR concentrations, which could be attributed to the abundance of free and unoccupied adsorbent sites available for easily interaction with dye molecules. However, the elimination percentages decreased with increasing dye concentrations due to the saturation of almost adsorbent sites and the unavailability of more sites to adsorb the remaining dye molecules. A similar finding was documented by Luo et al.^74^ and Tamimi et al.^75^. Notably, all tested NPs exhibited higher affinity in the removal of MG than CR indicated by a higher removal rate. That could be ascribed to the electrostatic interaction of MG cationic groups with negatively charged NPs. Contrariwise, the lower affinity of tested NPs to anionic dye could be due to coulombic repulsion^76^. Additionally, the number of functional groups, the presence of acid (SO_3_^−^) and basic (CH_3_N^−^) groups, geometry, size and the arrangement of dye molecules could probably influence the dye adsorption capacity of NPs^77^. Furthermore, as noticed previously, the synergistic effect provided by FCFNCs improved the adsorptive performance for both dyes in a more satisfactory manner than FCNPs and FFNPs.

##### An exploratory adsorption study by FTIR

To confirm the removal of MG and CR by NPs under investigation, FTIR study of adsorbed dye-NPs was performed. As shown in Supplementary Fig. S8a, b and c, pronounced peaks were observed at 438 cm^−1^, which corresponded to the binding of cationic ions of MG; the peaks at 1194 cm^−1^ and 1163 cm^−1^ for aromatic C–N stretching vibrations. The peaks at 1597 cm^−1^ indicated C=C stretching of the benzene rings. Also, the bands at 1447 cm^−1^ and 1458 cm^−1^ were attributed to scissoring vibration of –CH_2_^77,78^.

On the other hand, the vibrational modes for CR-treated samples exhibited absorption peaks at 1551, 1641 and 1643 cm^−1^, which distinguished stretching vibration band of the azo chromophore (–N=N) for the red azo dyes as stated by Bartošová et al.^72^. In addition, the presence of vibration bands at 1113, 1127 and 1180 cm^−1^, which characterized asymmetric stretching vibration of S–O(SO_3_-H) group might imply successful adsorption of CR on the surfaces of FCNPs, FFNPs and FCFNCs (Supplementary Fig. S9a, b and c). Notably, in the remediation profiles of both dyes, new unique bands were noticed including bands at wavelengths 884, 872 and 877 cm^−1^ for p-disubstituted benzene ring vibrations; 748 and 938 cm^−1^ for (=C–H) of aromatic rings^78^. Furthermore, there were shifts in some peak’s positions, especially of Cu and Fe in FCNPs and FFNPs and FCFNCs, with slight alterations in their intensities. Such observations could have several explanations, including a sufficient interaction between functional groups of the dye’s molecules with NPs, cleavage of some functional groups of dyes (e.g. amine) and involvement of new functionalities in the adsorption of dyes on the surface of NPs via Van der Waals forces or weak electrostatic interaction^73,78,79^.

##### Toxicity analysis

The crude untreated and NPs-treated MG and CR solutions (200 mg/L and 500 mg L^−1^) were subjected to microbial toxicity assay versus different microbial classes such as *Proteus mirabilis, Bacillus mojavensis*, and *Metschnikowia pulcherrima* (Supplementary Fig. S10). Initially, the control cultures (on NB and Sabouraud dextrose media) exhibited considerable growth by 1.46 × 10^9^, 9.85 × 10^8^ and 1.52 × 10^9^ CFU/mL for *P. mirabilis, B. mojavensis*, and *M. pulcherrima*, respectively. While, untreated crude MG solutions of 200 mg/L and 500 mg/L concentrations caused growth reduction of 60.62 ± 2.5% and 83.18 ± 1.7% in *P. mirabilis*; 49.39 ± 1.3% and 60.68 ± 1.3% in *B. mojavensis*; and 39.95 ± 1.1 and 62.61 ± 1.8% in *M. pulcherrima,* respectively*.* Regarding CR (200 mg/L and 500 mg/L) the results showed microbial growth suppression by (50.08 ± 2.1, 68.1 ± 1.3%), (42.16 ± 2.5, 60.93 ± 1.5%) and (56.74 ± 2.5, 71.73 ± 0.8%) for *P. mirabilis, B. mojavensis*, and *M. pulcherrima*, correspondingly. Through FCNPs, FFNPs and CuFe-FCFNCs treatments, an improvement of microbial growth was noticed and inhibition percentage reduced to the range of 9–12% and 20–26% for 200 mg/L and 500 mg/L of MG, respectively. In the case of CR-treated solutions containing 200 mg/L and 500 mg/L, the inhibition percentages recorded 15–20% and 28–30%, respectively.

For cytotoxicity assessment of MG dye, the viability of Wi-38 and Vero human cells diminished to 54.16–58.49% and 49.78–52.54% after incubation with 200 mg/L and 500 mg/mL of MG, respectively (Supplementary Fig. S11a and b). However, incubation of these cells with 1 g/L of FCFNCs-treated MG solutions (200 mg/L and 500 mg/L) elevated viability of Wi-38 and Vero cells (p > 0.05) to (81.96, 85.38%) and (80.49, 81.64%), respectively. Regarding CR dye, after exposure to 200 mg/mL, the viability of Wi-38 and Vero cells decreased to 54.61% and 58.98%, respectively. A 500 mg/mL of the dye lowered the viability of Wi-38 and Vero cells to 50.38% and 54.81%, respectively. As depicted in Supplementary Fig. S11c and d, FCFNCs significantly suppressed the CR cytotoxicity, which was confirmed by the highest viability of Wi-38 and Vero cells by 94.87–98.82% at 200 mg/mL and 84.52–88.01% at 500 mg/mL, respectively.

From our perspective, the mutual effect of combined NPs in FCFNCs, which imitated the antibiotic’s effect and whatever the mechanism of antimicrobial action, showed their potential for application in the adjuvant or combination therapy to defeat pathogens that exhibit multidrug-resistance capability. Besides, the safety level offered by FCFNCs could permit their utilization in agriculture, prevention of biofouling, food packaging, anti-corrosion for water pipes and in pollution remediation instead of rare and expensive noble metals such as Ag, Au, Pt and Pd. The anticancer activity exhibited by our hybrid nanocomposite promotes its application medically in preventing infections, wounds-healing and in contraceptive devices and bone prostheses. Furthermore, the powerful remediation capacity of FCFNCs observed with MG and CR encourages its recruitment in detoxification of effluents from the textile industry, before discharging into water bodies, which represent more than 15% of water pollution as mentioned by Ismail et al.^70^.

## Conclusion

To sum up, low cost, adaptive physicochemical properties and their compatibility with biological materials are the main reasons for selecting Cu and Fe in hybridization process to synthesize their binary hybridized nanoform. In specific, designing a synthesis strategy that is biosafe, biocompatible and environmentally benign is a challenge. Herein, *S. cyaneofuscatus* EM3 was invested as bionanofactory for the biofabrication of FCFNCs, for the first time. The structural properties of as-synthesized FCFNCs were determined through, XRD, EDX, elemental mapping, FTIR, UV–Vis spectroscopy, TEM, and ζ-potential. The highest EC_100_ values of FCFNCs and the strongest apoptosis-dependent anticancer activity against Caco-2, HepG-2, MCF-7 and PC-63 implying the suitability and efficiency of FCFNCs in therapeutic and biomedical applications. Comparing to their single nanoforms, the biosynthesized FCFNCs demonstrated significant antimicrobial activity in the following order: *B. cereus ˃ S. aureus ˃ E. faecalis ˃ E. coli ˃ S. typhi ˃ P. aeruginosa*. Besides, FCFNCs in 250 µg mL^−1^ and 500 µg mL^−1^ concentrations demonstrated significant (*P *˂ 0.05) anticandidal action by achieving, respectively, 52% and 77.5% inhibition of *C. albicans.* However, the antibiofilm activity recorded 90.6 ± 1.3 and 76.11 ± 1.17% inhibition for *S. aureus* and *P. aeruginosa*, respectively. Also, the disintegration potency of FCFNCs at 750 µg mL^−1^ versus preformed *S. aureus* and *P. aeruginosa* biofilms reached 58.8 ± 0.7 and 41.8 ± 0.7%, respectively. Furthermore, the capability of FCFNCs to remediate both cationic (MG) and anionic (CR) dyes in 180 min revealed the prospective employing in the treatment of dye-laden textile effluents. Generally, the collective properties of biologically produced FCFNCs open up their applications in different scientific and technological fields.

## Materials and methods

### Microorganism, cultural conditions and biosynthesis method

From a sediment sample collected from Marriott Lake, basin number 3, Alexandria, Egypt, a bacterial isolate designated EM3 was selected. It was one of the existing indoor isolates exhibiting versatile biochemical and physiological capabilities. For molecular identification, the 16S rDNA sequencing technique was employed; the data of sequencing identified it as *S. cyaneofuscatus* EM3. Its 16S rDNA sequence was deposited in the GenBank database under the accession number KY964508. The spore suspension was maintained in 20% (v/v) glycerol at − 20 °C for subsequent use. For NPs synthesis, a bacterium lawn composed of 5 discs (9 mm diameter) taken from 72 h culture was inoculated in 250 mL Erlenmeyer flasks containing 70 mL of starch-nitrate broth^7^ supplemented with 1 mM of each Fe(NO_3_)_3_·9H_2_O, Cu(NO_3_)_2_ and both. The inoculated cultures were incubated for 96 h at 30 °C while being shaken orbitally at 150 rpm. According to the minimal inhibitory concentration (MIC) test (data not shown), the examined concentration (1 mM) was selected. Meanwhile, two control samples, i.e., media containing bacteria without metal precursors and media containing metal precursors without bacteria, were incubated exactly as the test flasks. The sign of NPs synthesis was detected visually during the incubation period through changes in media and biomass colors. At the end of incubation, the bacterial biomass containing NPs were harvested by centrifugation at 10,000×*g* for 20 min and washed three times with doubled distilled H_2_O to remove media residues; followed by NPs extraction via a protocol described by Eltarahony et al.^4^ with slight modification. The bacterial suspension in the lysis buffer was sonicated for 5 min at 60–80% amplitude and frequency of 20 kHz with 0.6 s pulse rate to ensure complete disruption of Streptomyces pellets. The lysed suspension was subjected to a vigorous vortex for 5 min to obtain a homogeneous mixture. The extracted NPs were analyzed for characterization after centrifugation, washing and drying^2^.

### Physicochemical characterization of NPs

#### Structural characteristics

The identification of crystal phase and NPs purity were acquired using Bruker MeaSrv (D2-208219) X-ray diffractometer with Cu Kα radiation tube (λ = 1.5406 Å) operating at a voltage of 30 kV, an electric current of 30 mA and scanned between 2θ angular range of 2° to 100° at a scan speed of 0.02°/s. Then, JCPDS database was used to analyze the data^80^.

#### Compositional characteristics

The electron localization on the materials was examined by the electron mapping function of the scanning electron microscope. Also, empirical elemental analysis (qualitative and semiquantitative analysis) of NPs were pursued using EDX, JEOL JEM-1230, Japan^80^.

#### Functional characteristics

The active chemical bonds or functional groups conjugated with NPs were determined by Shimadzu FTIR-8400 S, Japan. Disc method using KBr was employed as a matrix; the spectrum was scanned between 4700 and 400 cm^−1^ at a resolution of 4 cm^−1^.

#### Optical characteristics

The optical properties of the as-prepared NPs were primly determined through UV–Visible spectroscopy (Labomed model-double beam) in a wavelength range of 200–800 nm at room temperature. The SPR of the electrons on the surface of the metal NPs exhibit tunable and unique peak at the precise wavelengths.

#### Morphological characteristics

The morphology and size of NPs were visualized by (TEM), JEOL JEM-1230-Japan, operated at 200 kV^80^.

#### Surface charge characteristics

The hydrodynamic size and ζ-potential of the biosynthesized NPs were measured by Zetasizer Nano ZS (Malvern Instruments, Worcestershire). The measurements were carried out at 25 °C, at a fixed scatter angle of 173° and the data were analyzed by Zetasizer software 6.

### Application of as-prepared NPs

#### MTT assay for determination of cytotoxicity on human normal and cancer cell lines

The cytotoxicity of the biosynthesized FCNPs, FFNPs and FCFNCs was determined using normal human lung fibroblast Wi-38 cells. Wi-38 cell line was cultured in 10% fetal bovine serum (FBS)-contained DMEM medium, seeded with 5 × 10^3^ cells per well in a 96-well cell culture plate and incubated in 5% CO_2_ incubator at 37 °C. After 24 h of the cell attachment, serial concentrations of the tested NPs were incubated with Wi-38 cells for 72 h. Cell viability was assayed by MTT method^81^. Twenty microliters of 5 mg/mL MTT (Sigma, USA) were added and the plate was incubated at 37 °C for 3 h. Then MTT solution was removed, 100 µL DMSO was added and the absorbance was measured with a microplate reader (BMG LabTech, Germany) at 570 nm. The concentrations of the tested NPs which cause 100% cell viability (EC_100_) were estimated by the Graphpad Instat software.

The anticancer effect of the above-mentioned treatments was assayed using four human cancer cell lines. Colon cancer cells (Caco-2) were maintained in DMEM (Lonza, USA) containing 10% FBS, while liver (HepG-2), breast (MCF-7) and prostate (PC-3) cancer cell lines were cultured in RPMI-1640 (Lonza, USA) supplemented with 10% FBS. All cancer cells (5 × 10^3^ cells/well) were seeded in a sterile 96-well plates. After 24 h, serial concentrations of the tested nanomaterials were incubated with cancer cell lines for 72 h at 37 °C in a 5% CO_2_ incubator. MTT method described above was followed. The half maximal inhibitory concentration values for cancer growth (IC_50_) were calculated using the Graphpad Instat software. Furthermore, cellular morphological changes before and after treatment were inspected using phase-contrast inverted microscope with a digital camera (Olympus, Japan). Additionally, the synergy between Cu and Fe in the hybrid nanocomposite, in the term of IC_50_ values, was investigated by estimating of the combination index (CI), according to Long et al.^82^.

#### Flow cytometric analysis of apoptosis-induced anticancer effect of FCFNCs

Briefly, the studied NPs were added to the attached Caco-2, HepG2, MCF-7 and PC-3 cell lines. After 72 h incubation and cell trypsinization, the untreated and treated cells were incubated with fluorescein isothiocyanate (FITC)-annexin V/PI for 15 min. Thereafter, the apoptosis-dependent anticancer potential was assessed by the quantification of annexin-stained apoptotic cells using the FITC signal (FL1) detector against the phycoerythrin emission signal (FL2) detector^83^. The synergy between Cu and Fe in their hybrid FCFNCs was investigated as CI that is the ratio of the average of the excepted values of total apoptosis between the individual NPs and observed values in hybrid nanocomposite (CI < 1, > 1 and = 1 denote a synergistic, an antagonistic and an additive effect, respectively).

#### Fluorescence microscope investigating apoptotic cells

In brief, the untreated and FCNPs-, FFNPs- and FCFNCs-treated cancer cells were stained with double nuclear dye consisting of 100 µg/mL ethidium bromide and 100 µg/mL acridine orange. After cell washing with phosphate buffer saline (PBS), apoptotic cells were observed under the fluorescent phase contrast microscope (Olympus, Japan).

#### Quantitative detection of fold change in the expression levels of tumor suppressor gene (p53) and caspase 3

Total RNAs of untreated, FCNPs, FFNPs and FCFNCs-treated HepG-2 cells were extracted then cDNAs were synthesized using Gene JET RNA Purification Kit (Thermo Scientific, USA) and cDNA Synthesis Kit (Thermo Scientific, USA), respectively. Real time PCR was performed using SYBR green master mix and specific primers (Forward/Reverse) were 5′-TAACAGTTCCTGCATGGGCGGC-3′; 5′-AGGACAGGCACAAACACGCACC-3′ for p53 gene and 5′-TTTGTTTGTGTGCTTCTGAGCC-3′; 5′-ATTCTGTTGCCACCTTTCGG-3′ for caspase 3 gene. The 2^−ΔΔCT^ equation was used to estimate the change in gene expressions of p53 and caspase 3 before and after treatment of the cancer cells. Also, CI was calculated as a ratio of the excepted and observed values.

#### Immunohistochemical expression of proliferation marker Ki-67

In summary, the untreated and FCNPs, FFNPs and FCFNCs-treated HepG2 cells were collected by trypsinization then washed, fixed, dehydrated and immersed in xylene before being impregnated in melted paraffin to form solid paraffin blocks. These blocks were cut into micro-thick sections and loaded into positively charged slides. Slides were dried, dewaxed, rehydrated and incubated in H_2_O_2_ for 10 min. After slide washing with PBS buffer, placing in 10 mM citrate buffer and heating for 10–20 min, the cold slides were soaked overnight in primary Ki-67 antibody before being processed according to the manual protocol of the Ki-67 immunohistochemistry specific kit. The cellSens imaging analysis software of the phase-contrast microscope was used to evaluate the percentage of immunostained cells^84^.

##### Investigation of prooxidant effect-mediated anticancer activity by determining the intracellular reactive oxygen species (ROS) and the oxidized glutathione (GSSG) levels

The intracellular ROS level was examined by incubation of FCNPs, FFNPs and FCFNCs with HepG-2 cell line in a 5% CO_2_ incubator. After 72 h, the untreated and treated cancer cells were incubated with 5 μM of dichloro-dihydro-fluorescein diacetate (DCFH-DA) for 30 min at 37 °C in the dark. Then cells were trypsinized and suspended in a fresh PBS. The intensity of the fluorescence oxidized form of DCFH-DA was analyzed by flow cytometer with excitation and emission wavelengths at 488 nm and 530 nm, respectively^85^. Further, CI was calculated as above.

Regarding GSSG level, the cell lysates of the untreated and NPs-treated HepG-2 cells were incubated with L-vinyl-pyridine for 2 h. After that, 50 U/mL of GSH reductase, 0.3 mM NADPH and 6 mM 5,5′-dithiobis-(2-nitrobenzoic acid) for 10 min at 30 °C. Then the rate of absorbance at 412 nm was measured at 0 and 5 min using a microplate reader (BMG LabTech, Germany) at 570 nm to quantify the level of GSSG according to Griffith's method^86^ with the modification of Tietze's assay^87^. Additionally, CI was estimated as above.

#### Antimicrobial activity

The antagonistic activity of FCNPs, FFNPs and FCFNCs was evaluated by turbidity assay, which provides better diffusion and contact with examined microbes as recommended by Pinto et al.^22^ and Zare et al.^88^. About 1 × 10^8^ CFU/mL of Gram-negative bacteria [*Escherichia coli* (ATCC 25922), *Salmonella typhimurium* (ATCC 14028), *Pseudomonas aeruginosa* (ATCC 15442)], Gram-positive bacteria [*Bacillus cereus* (ATCC 33019), *Enterococcus faecalis* (ATCC 29212), *Staphylococcus aureus* (ATCC 29213)] and yeast [*Candida albicans* (ATCC 10231)] were inoculated into 30 mL nutrient broth (bacteria) and Sabouraud dextrose broth (yeast) in the presence of 250, 500 and 750 µg/mL of NPs. Comparatively, the reference antibiotics tetracycline (for bacteria) and nystatin (for yeast) were used. The inoculated flasks were incubated under aerobic conditions with shaking at 150 rpm at 30 and 25 °C for 24 h and 48 h for bacteria and yeast, respectively. In comparison, two controls were simultaneously examined, positive controls containing microbial culture devoid of NPs and negative control containing NPs without microbial culture. The optical density (OD) at 600 nm was measured as a function of microbial growth/inhibition. The antagonistic efficiency of each treatments was calculated by the Eq. (16)^4^

16 $$ {\text{The}}\;{\text{ antagonistic}}\;{\text{ efficiency}} = \, ({\text{A}} - {\text{A}}_{{\text{o}}} )/{\text{ A }} \times \, 100 $$

where A indicates the absorbance of the positive control and A_0_ represents the absorbance of the samples treated with an antimicrobial agent. The experiment was carried out in triplicates and the data were expressed as means ± SEM (standard error of mean).

#### Antibiofilm activity

##### Biofilm synthesis assay

The microtitre plate method was employed as a standard assay for detecting the biofilm synthesis in the coexistence of 500 and 750 µg/mL of FCNPs, FFNPs, FCFNCs and tetracycline. Briefly, 100 μL of LB medium (Sigma-Aldrich) containing 10^8^ CFU/mL of *P. aeruginosa* (Gram-negative) and *S. aureus* (Gram-positive) were seeded into the wells of a sterile polystyrene microtitre plate. Meanwhile, the controls comprising bacterial cultures without NPs were assessed. The plates were incubated for 24 h under static incubation at 37 °C. In order to remove planktonic cells, the plates were emptied, washed thrice with phosphate buffer saline (PBS) and stained with 200 μL of (0.1%, w/v) crystal violet for 30 min at 37 °C. Thereafter, washing, fixation and solubilization of stained biofilm by 95% ethanol (200 μL) were performed. Finally, total biofilm formation and inhibition were spectrophotometrically assayed at 595 nm. All the treated and untreated samples were conducted in triplicate and the results were expressed as mean ± SEM^2^. The previous Eq. (16) was utilized to calculate an inhibition percentage of biofilm synthesis.

##### Detachment assay of established biofilm

The potency of FCNPs, FFNPs, FCFNCs and tetracycline for disaggregating the already formed biofilms by *P. aeruginosa* and *S. aureus* were evaluated. Initially, the bacterial cultures adjusted to an OD_600_ of 0.1 (0.5 McFarland standard) were inoculated into 96-well microplates and incubated at 37 °C statically for 24 h to allow biofilm construction. Subsequently, 500 and 750 µg/mL of FCNPs, FFNPs, FCFNCs and tetracycline were added under aseptic conditions to each well. The incubation, processing, staining and fixation were carried out as described formerly. The disintegration percentage of the biofilms was calculated using Eq. (16). All the experiments were carried out in triplicate and the results are expressed as mean ± SEM.

#### Algicidal activity

Against the unicellular photosynthetic *Chlorella vulgaris*, the algicidal impact of 500 and 750 µg/mL of FCNPs, FFNPs, FCFNCs and nystatin were investigated. As described previously by Gong et al.^89^, propagation of algae was performed in 250 mL Erlenmeyer flasks containing sterilized Bold’s basal medium (BBM). Incubation was carried out at 25 °C under illumination following the daily cycles of 12 h light and 12 h night for seven days. The positive control without NPs treatment was run in parallel. The cells were quantified by a hemocytometer under a light microscope (Olympus BH-2, Japan). The inhibition percentage was calculated by Eq. (16), and the results are expressed as means ± SEM.

#### Dye detoxification

The removal efficiency of FCNPs, FFNPs and FCFNCs for 50, 200 and 500 mg/L of Congo red (CR, C_32_H_22_N_6_Na_2_O_6_S_2_) and Malachite green (MG, C_23_H_25_N_2_) dyes was executed by batch mode system. In orbital shaker at constant agitation speed (150 rpm) and constant temperature (30 °C), a fixed amount of FCNPs, FFNPs and FCFNCs (1 g/L) was added to 100 mL of different dyes concentrations (50, 200, 500 mg/L) and pH of the solutions was adjusted to 7 ± 0.2 with 0.1 N HCl and 0.1 N NaOH. At fixed time of incubation, 2 mL of treated and untreated dyes solutions were withdrawn and centrifuged (5000×*g* for 10 min). The treated supernatant was analyzed to determine the residual dye concentration by spectrophotometric measurement at 497 and 617 nm correspondingly, for CR and MG (T60 UV/VIS spectrophotometer). The experiments were conducted in duplicate and the controls without NPs treatments were simultaneously run. The decolorization percentage (D%) was calculated using Eq. (17)^70,73^^.^

17 $$ {\text{D}}\;\% = {\text{ C}}_{{\text{o}}} - {\text{C}}_{{\text{t}}} /{\text{ C}}_{{\text{o}}} \times \, 100\% $$

where C_o_ (mg/L) is the initial dye concentration and C_t_ (mg/L) is the final concentration of the dye at fixed time.

#### FTIR

FTIR analysis of dried NPs-adsorbed dyes was monitored following the procedure described previously.

##### Toxicity assays

The microtoxicity and cytotoxicity assays were carried out to evaluate the toxicity of dye solutions before and after NPs-treatment. Regarding microtoxicity, *P. mirabilis, B. mojavensis*, and *M. pulcherrima* were used as models of environmentally isolated prokaryotic and eukaryotic microbes. Nutrient agar and Sabouraud dextrose agar supplemented with dyes solution before and after treatment were utilized for cultivation of bacteria and yeast, respectively; incubated as recorded previously. As a negative control, the uninoculated dye solution was tested concurrently. The bacterial growth was detected and expressed in the terms of CFU/mL^73^.

However, the cytotoxicity assay was evaluated using human normal fetal lung cell line (Wi-38) and normal adult African green monkey kidney cell line (Vero) according to the method described by Mosmann^82^. Human Wi-38 and mammalian Vero cells were maintained in DMEM medium (Lonza, USA) containing 10% fetal bovine serum. These cell lines were sub-cultured for two weeks before assay using trypsin EDTA (Lonza, USA). After seeding and attachment of Wi-38 and Vero in 96 well cell culture plates, cells were exposed to solutions of untreated 200 and 500 mg/mL of CR and MG and treated with 1 g/L of each NPs type. After 72 h incubation in a 5% CO_2_ incubator, cell viability was detected by MTT assay as previously described.

### Statistical analysis

Data were represented as mean ± standard error of the mean (SEM). Statistical significance was determined by the multiple comparisons Tukey post-hoc analysis of variance (ANOVA) via the SPSS16 software. The differences were deemed statistically significant at p < 0.05.

## Supplementary Information

Supplementary Figures.

## Data Availability

All data generated or analyzed during this study are included in this article (and its Supplementary Information file).
