# Supplementary material for: Unveiling the role of novel biogenic functionalized CuFe hybrid nanocomposites in boosting anticancer, antimicrobial and biosorption activities
Source: Sci Rep. 2021 Apr 8;11:7790. doi: 10.1038/s41598-021-87363-z (PMC8032780; doi:10.1038/s41598-021-87363-z)
Supplement: Supplementary file 1 — Supplementary Figures. [file 41598_2021_87363_MOESM1_ESM.docx]

**Unveiling the role of novel biogenic functionalized CuFe hybrid nanocomposites in boosting anticancer, antimicrobial and biosorption activities**

Marwa Eltarahony^1^*, Marwa Abu-Serie^2^***,** Hesham Hamad^3^, Sahar Zaki^1^, Desouky Abd-El-Haleem^1^

^1^ Environmental Biotechnology Department, Genetic Engineering and Biotechnology Research Institute (GEBRI), City of Scientific Research and Technological Applications (SRTA-City), 21934, New Borg El-Arab City, Alexandria, Egypt

^2^ Medical Biotechnology Department, Genetic Engineering and Biotechnology Research Institute, (GEBRI), City of Scientific Research and Technological Applications (SRTA-City), 21934, New Borg El-Arab City, Alexandria, Egypt

^3^ Fabrication Technology Research Department, Advanced Technology and New Materials Research Institute (ATNMRI), City of Scientific Research and Technological Applications (SRTA-City), Alexandria, 21934, Egypt.

* **Corresponding author.**

**E-mail:** [m_eltarahony@yahoo.com](mailto:m_eltarahony@yahoo.com)  **(**Marwa Eltarahony**)**

[marwaelhedaia@gmail.com](mailto:marwaelhedaia@gmail.com) (Marwa Abu-Serie)

**Supplementary Figure S1:** JCPDS cards of Cu (a), Fe (b) and their oxides


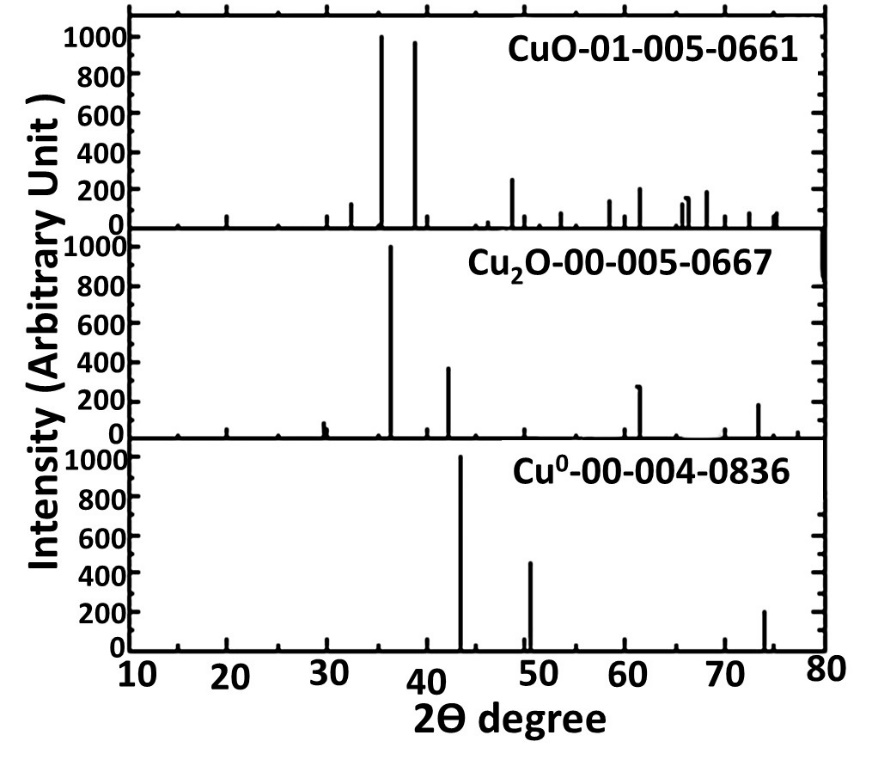

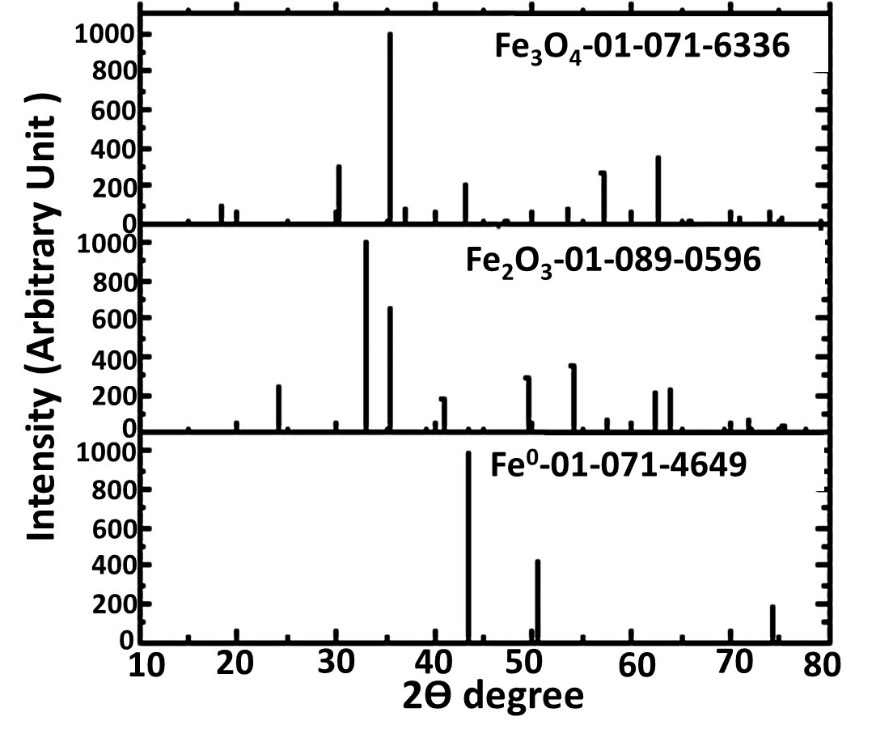


**a**

**b**

**Supplementary Figure S2:** Elemental mapping of Cu (**a**), O **(b**), Fe (**c**) in FCFNCs (**d**)


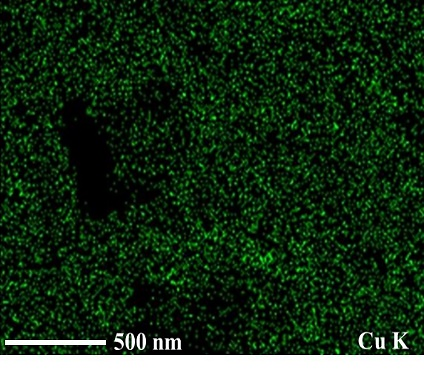


**a**


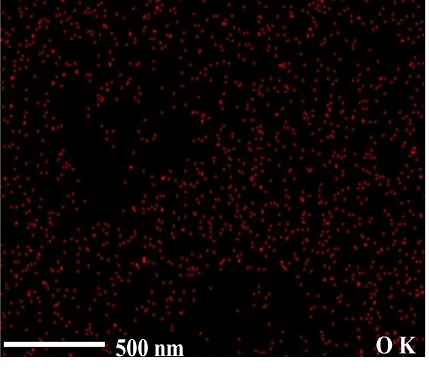


**b**


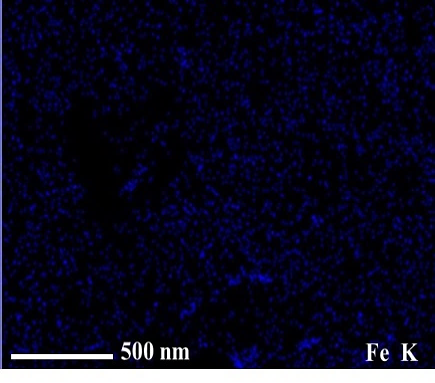


**c**


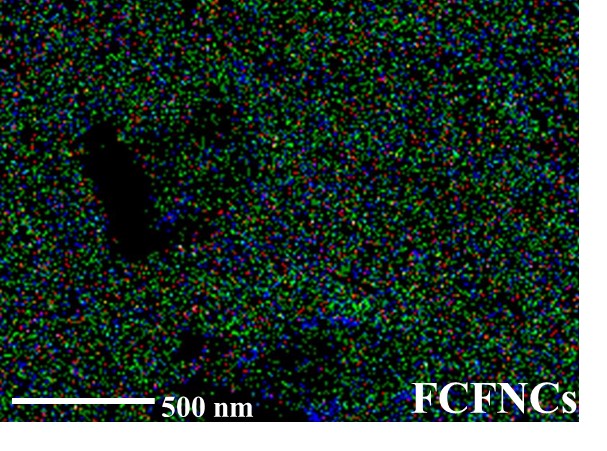


**d**

**Supplementary Figure S3:** EDX profiles of FCNPs (a) and FFNPs (b) synthesized by S. cyaneofuscatus EM3


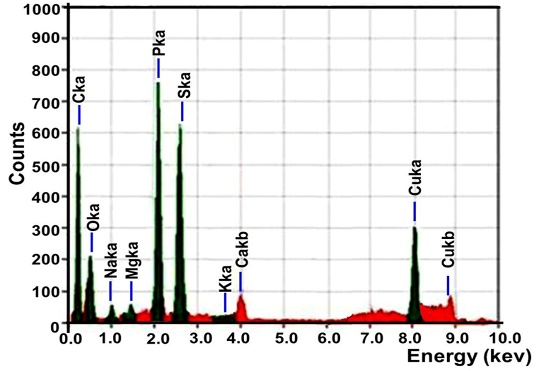


**a**


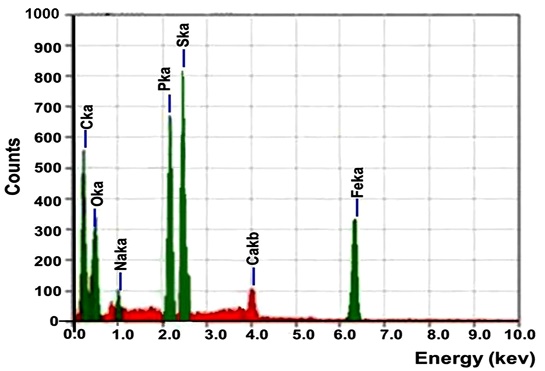


**b**

**Supplementary Figure S4:** TEM micrograph of cytoplasmic-localized FCNPs (a) and FFNPs (c) synthesized by Streptomyces cyaneofuscatus EM3 during stationary phase and after extraction (b) and (d), respectively


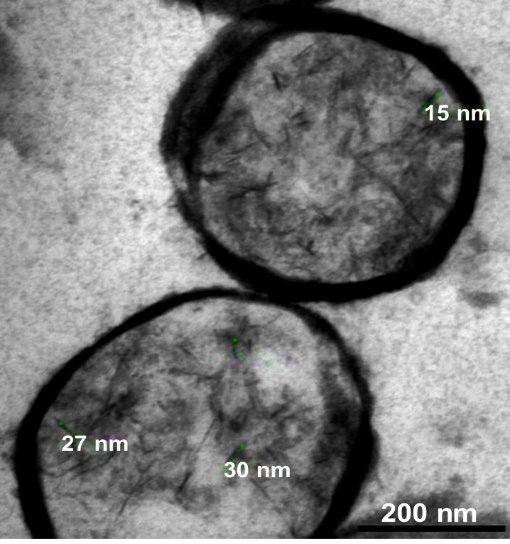


**a**


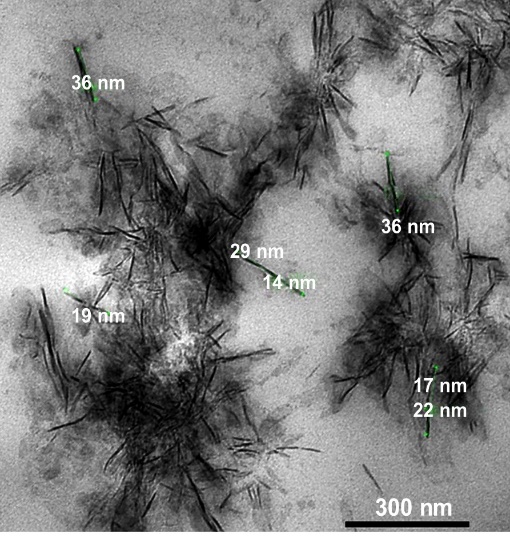


**b**


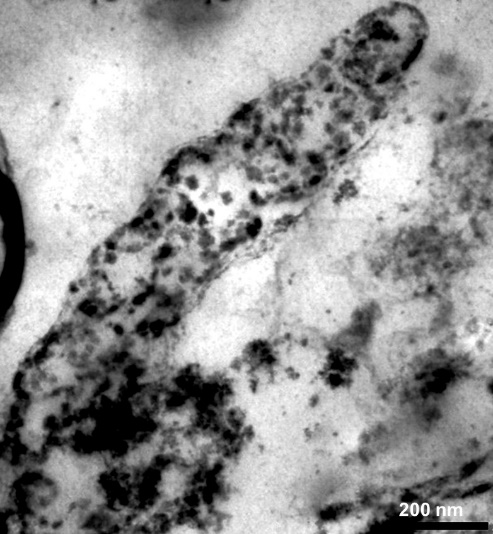


**c**


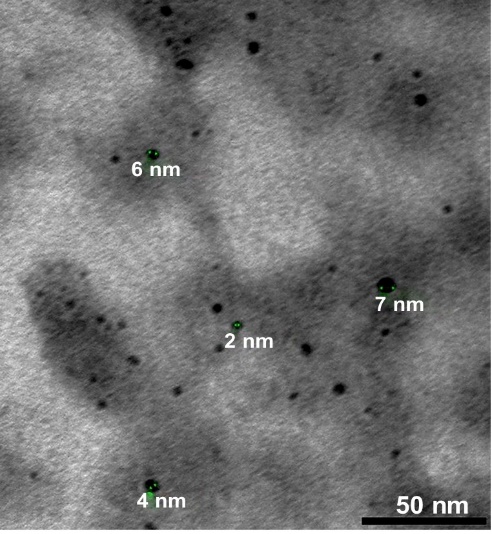


**d**

**Supplementary Figure S5:** Particle size distribution curve (a & b) and Zeta potential (c & d) of biosynthesized FCNPs and FFNPs, respectively


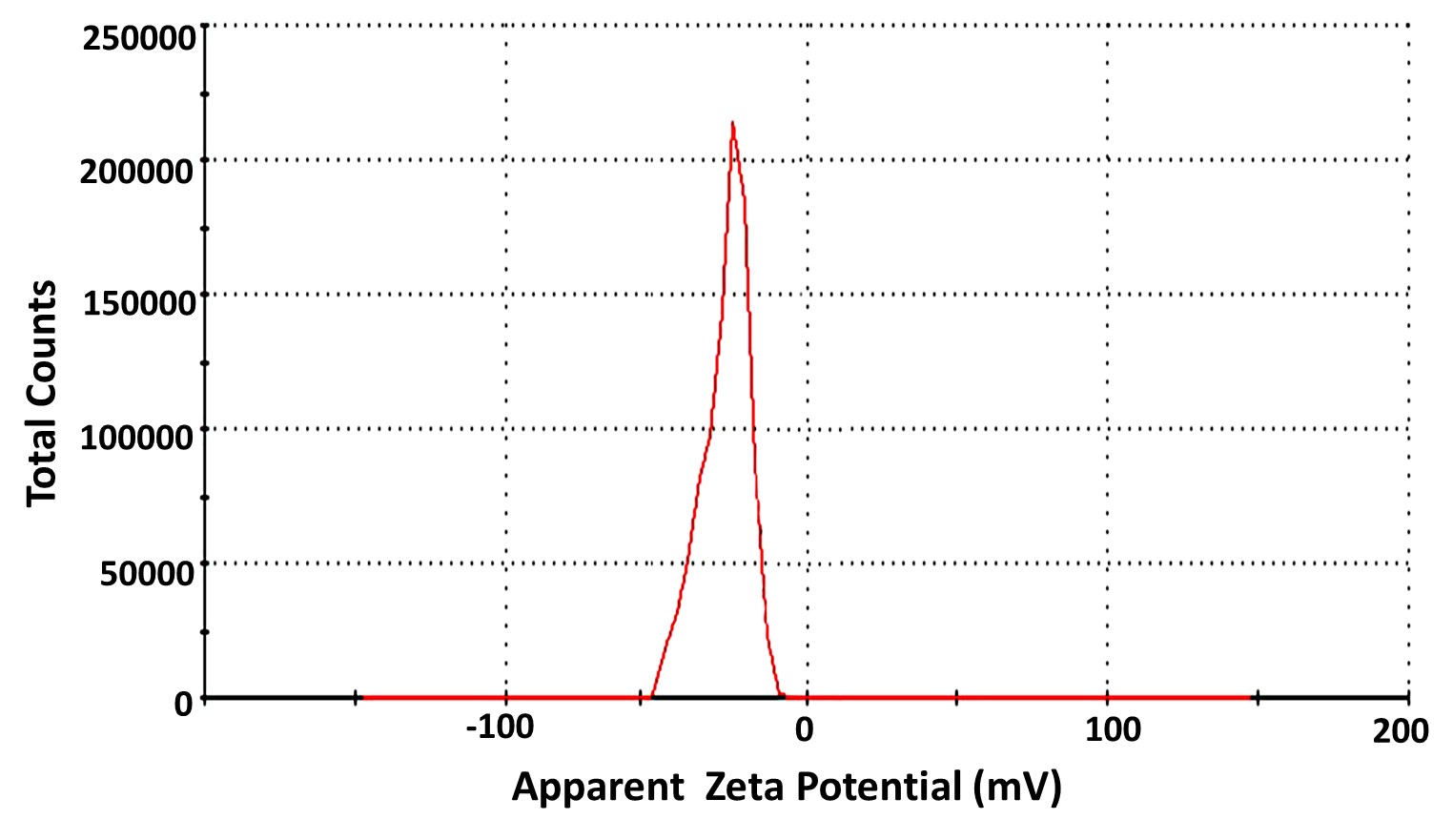

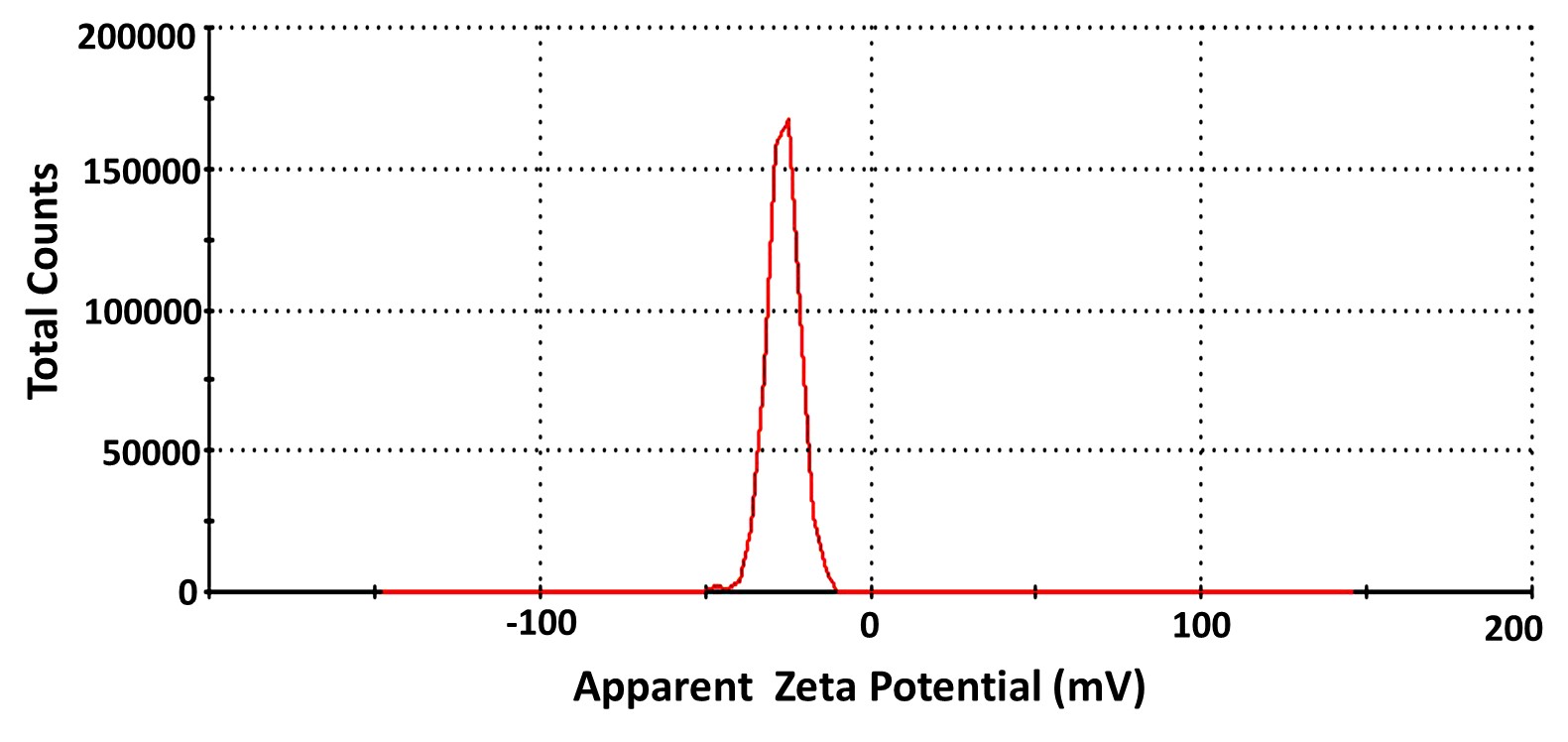

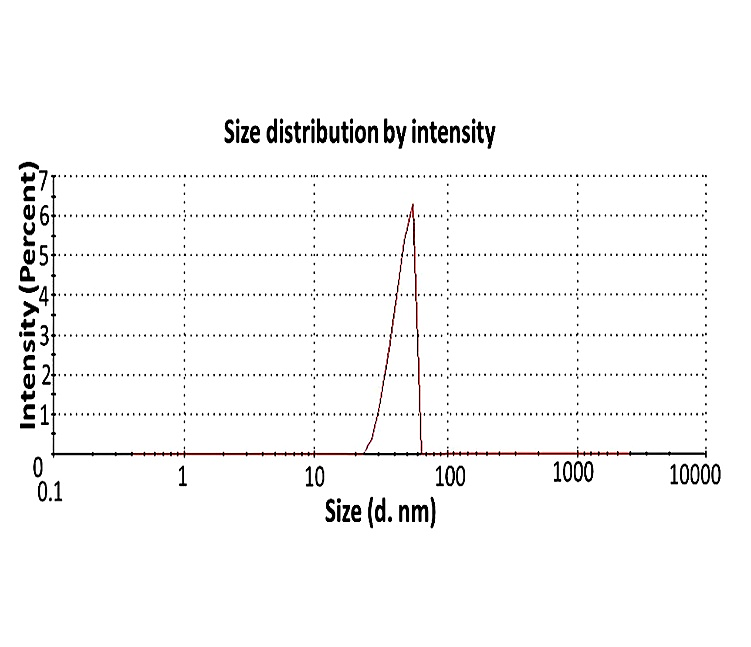


**a**


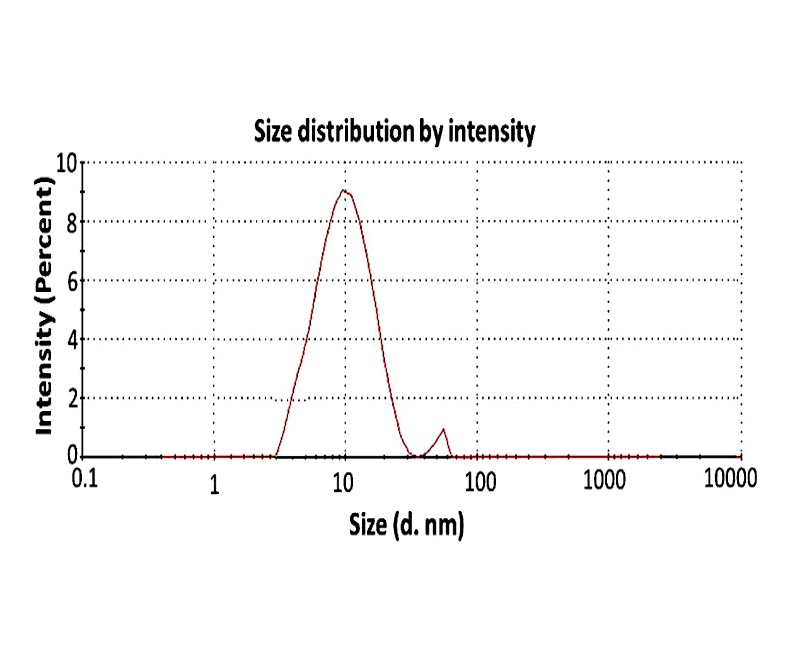


**d**

**c**

**b**

**Supplementary Figure S6:** Flow cytometric charts using annexin V/PI double staining with quantification of the % apoptotic cells in the control and treated cancer cells, all values were expressed as mean ± SEM


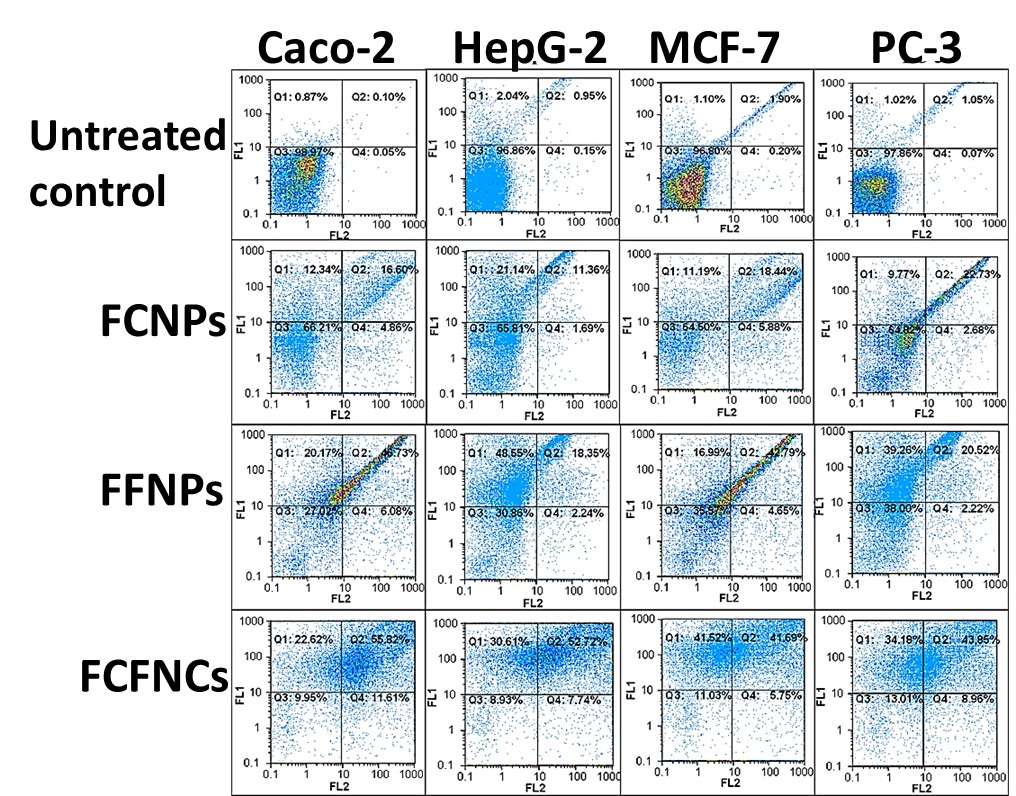


**Supplementary Figure S7:** Decolorization efficiency of different concentrations of MG and CR under constant NPs concentration (1 g/l), temperature (30 °C), pH 7 and agitation speed (150 rpm)

**
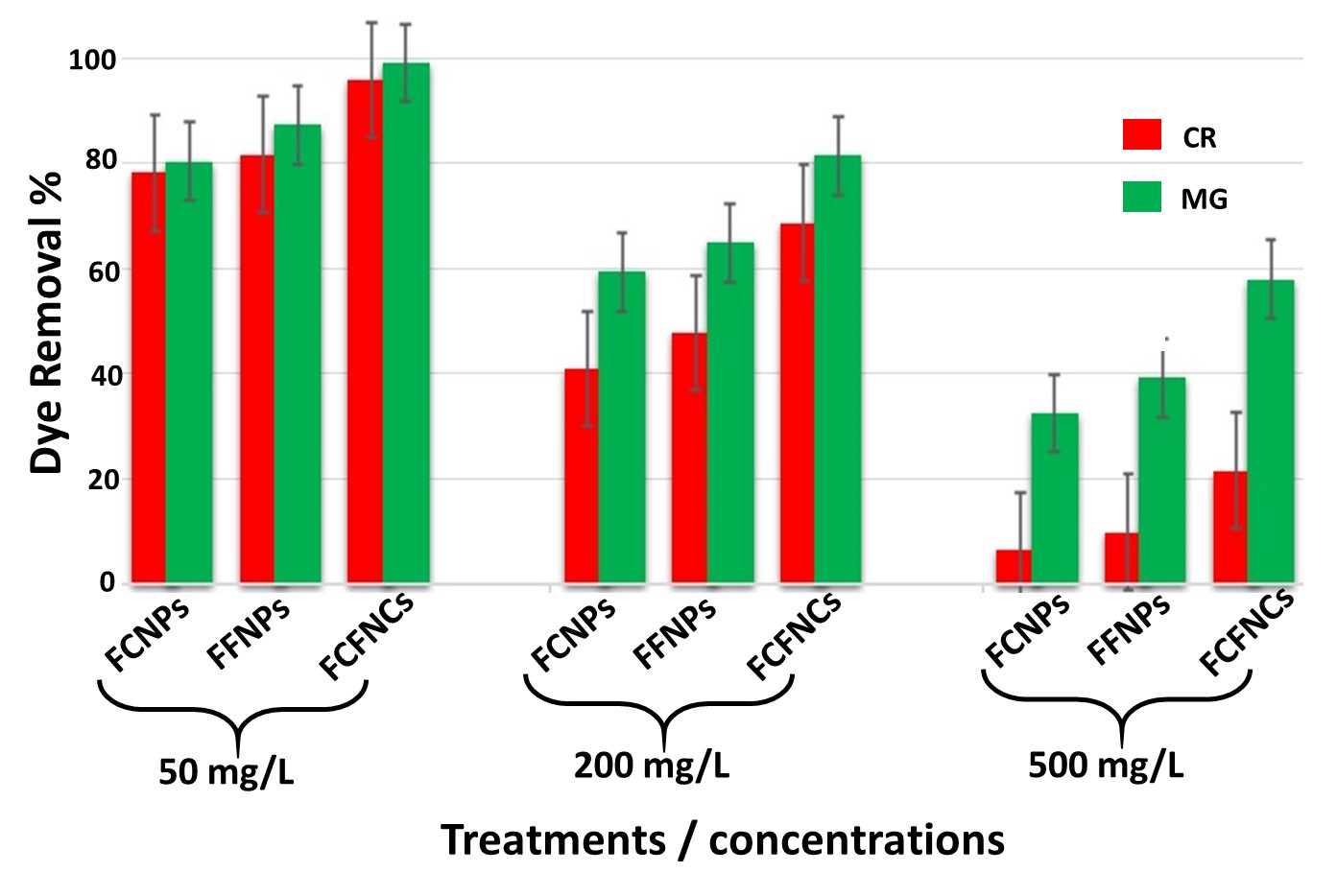
**

**Supplementary Figure S8:** FTIR spectra of NPs-adsorbents after removal of MG dye, a)- MG treated with FCNPs; b)- MG treated with FFNPs and c)- MG treated with FCFNCs


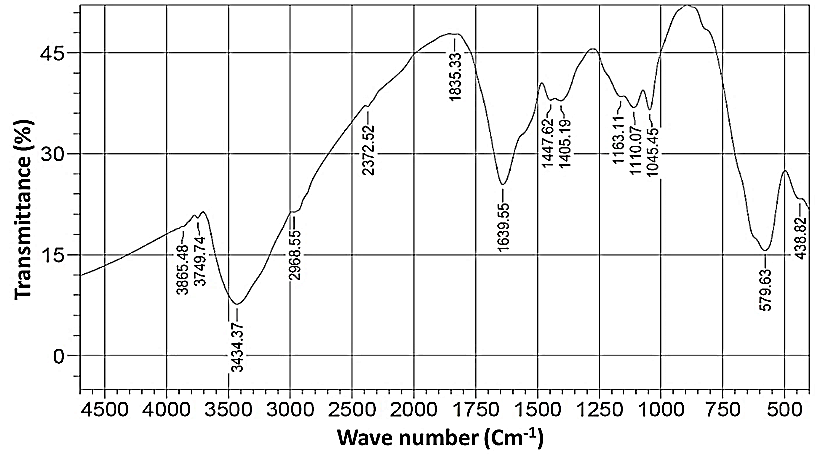


**a**


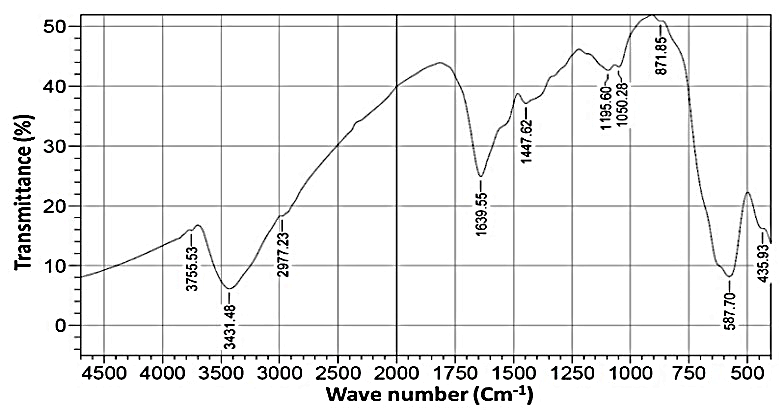


**b**


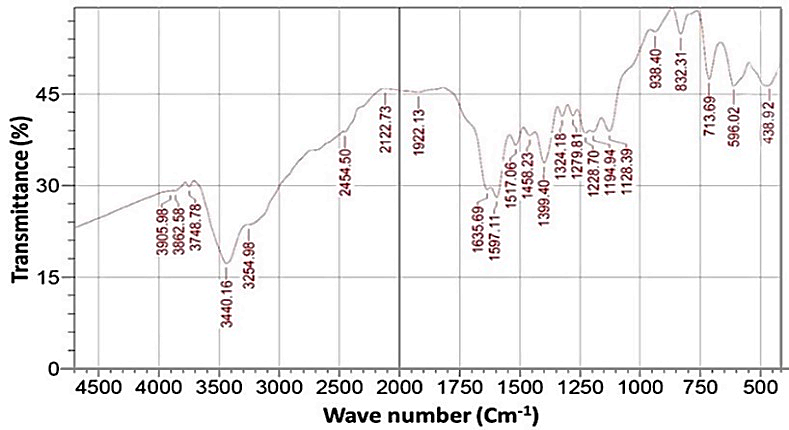


**c**

**Supplementary Figure S9:** FTIR spectra of NPs-adsorbents after removal of CR dye, a)- CR treated with FCNPs; b)- CR treated with FFNPs and c)- CR treated with FCFNCs


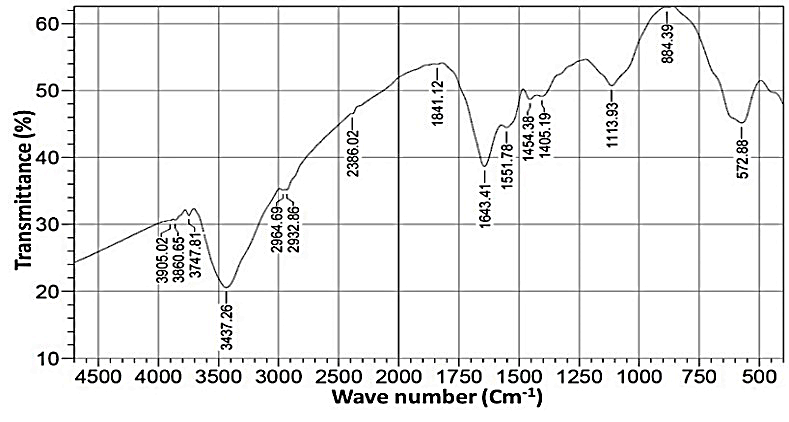


**a**


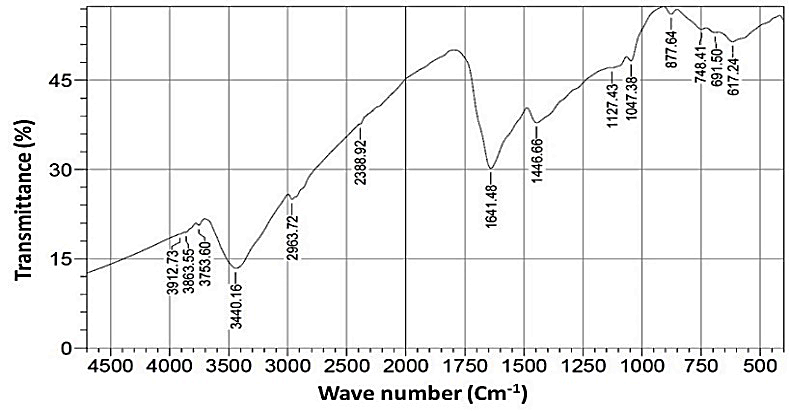


**b**


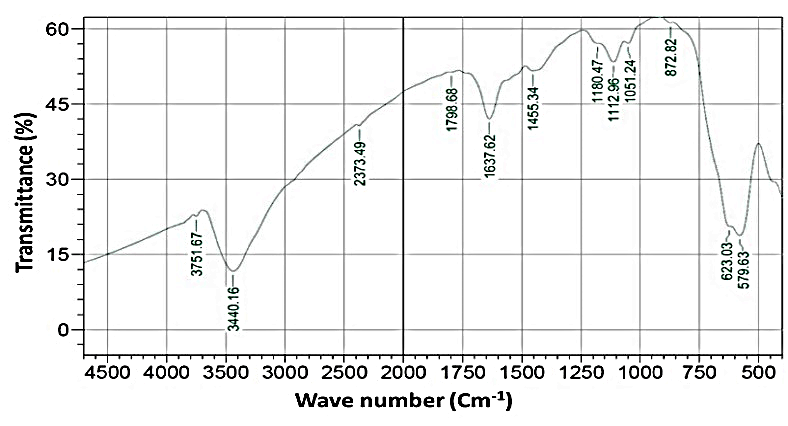


**c**

**Supplementary Figure S10:** Microtoxicity evaluation of MG (a) and CR (b) solutions after NPs-treatments pointing out to the viability percentages of *P. mirabilis,* *B. mojavensis*, and *M. pulcherrima*


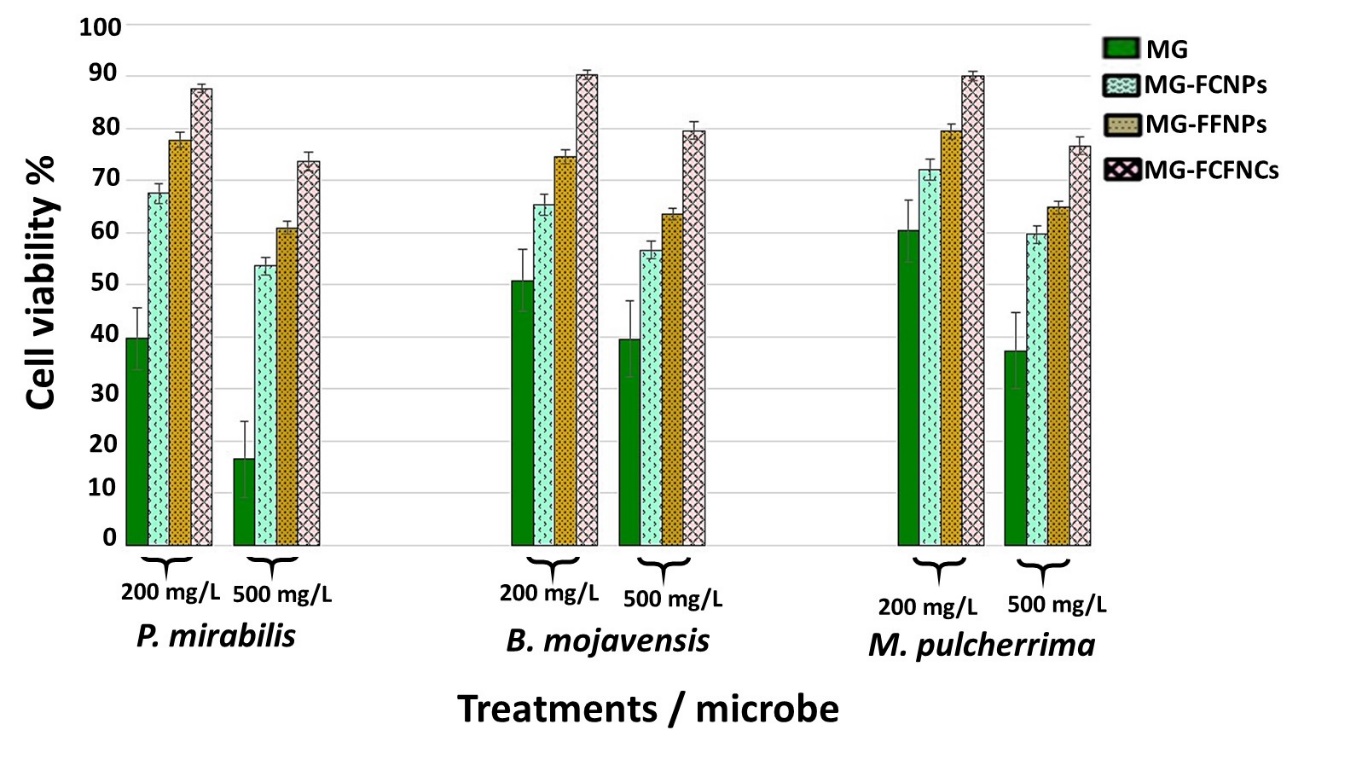


**a**


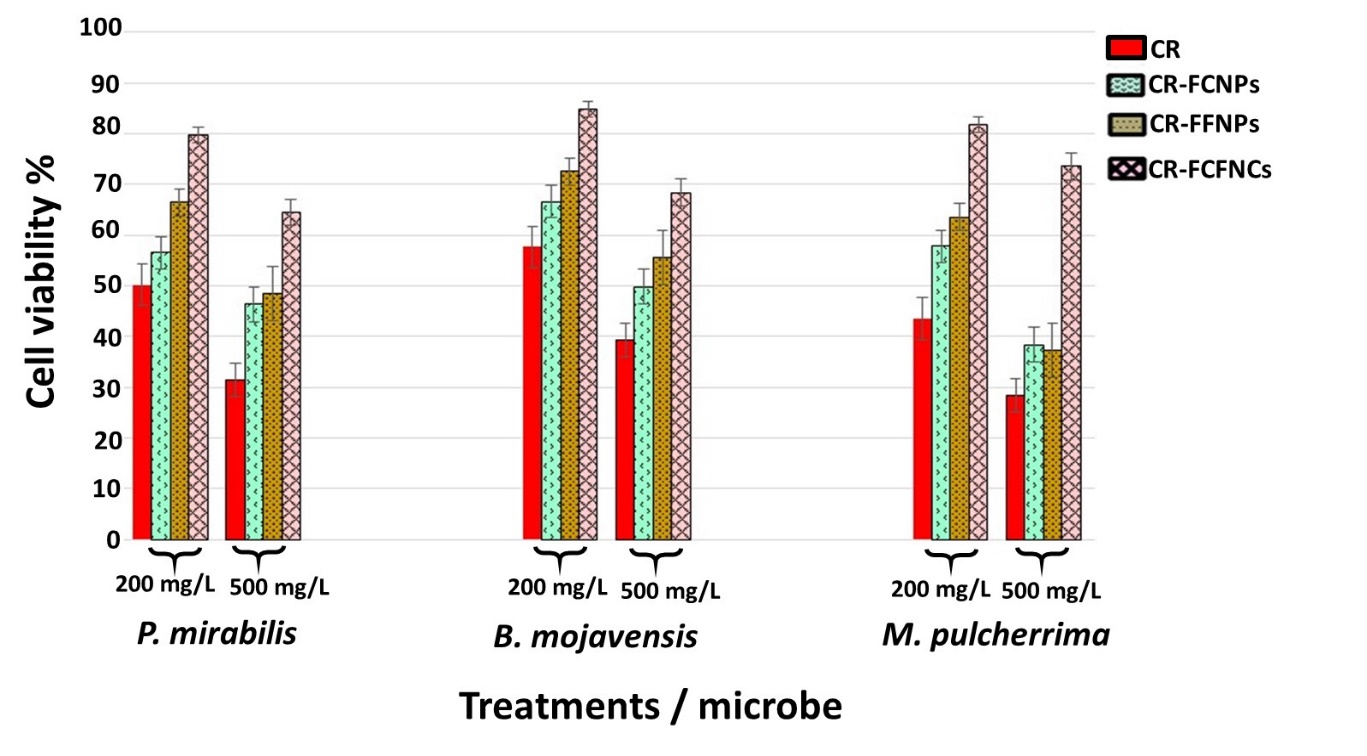


**b**

**a**

**Supplementary Figure S11:** Viability (%) of Wi-38 and Vero cells after 72 h exposure to 200 and 500 mg/ml MG (a &b) and CR dyes (c &d) with and without treatments of FCNPs, FFNPs and FCFNCs. All values were expressed as mean ± SEM. FCFNCs were compared with all other treatments with significance at *P*-value <0.05*, <0.005**, <0.0005***.


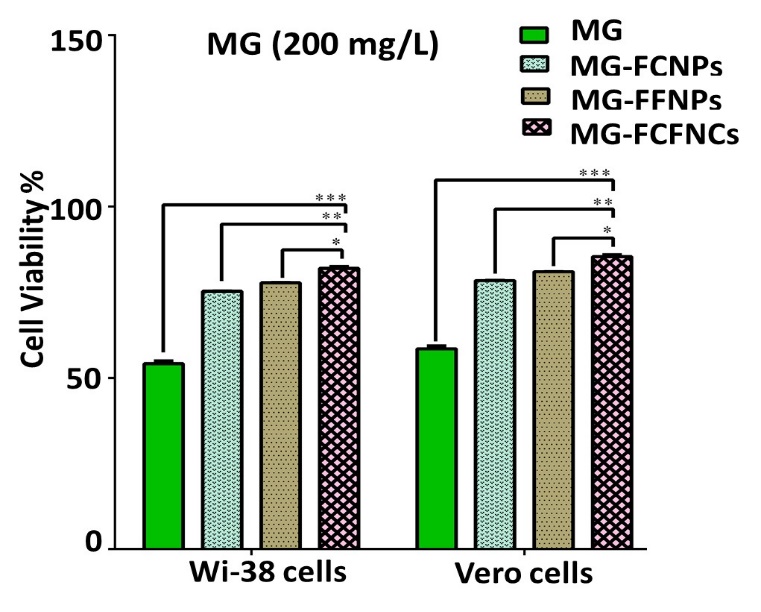


**a**


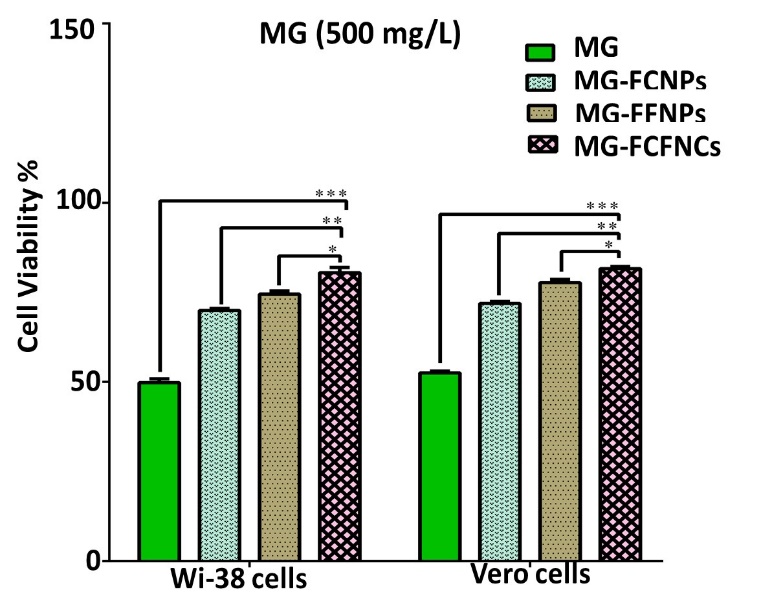


**b**


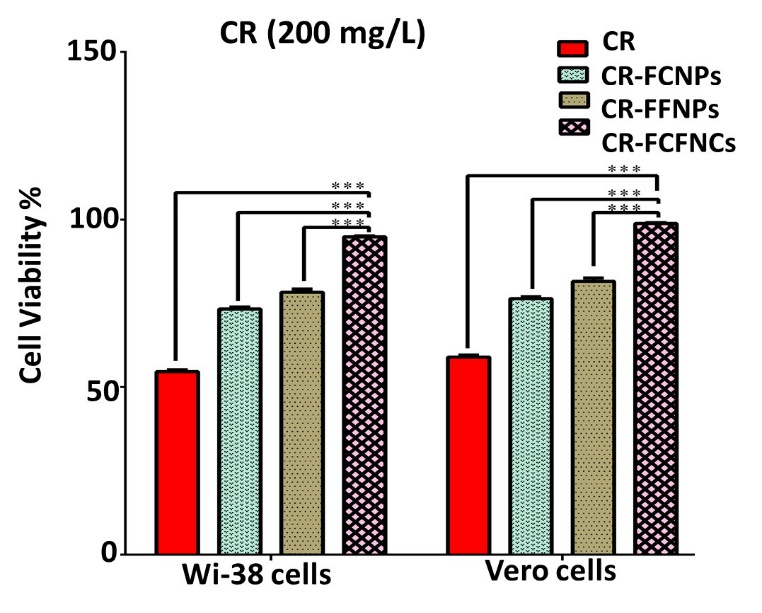


**c**


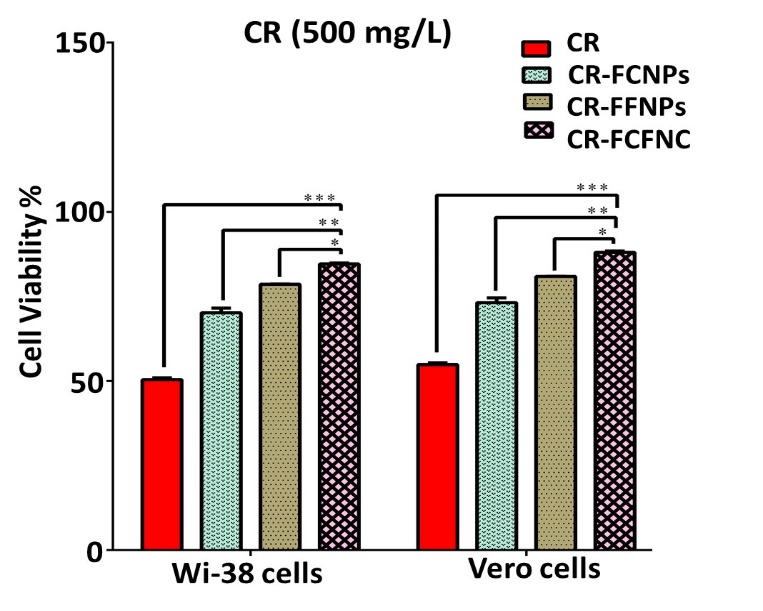


**d**
